# TREM2 in Neurodegenerative Diseases

**DOI:** 10.1186/s13024-017-0197-5

**Published:** 2017-08-02

**Authors:** Taylor R. Jay, Victoria E. von Saucken, Gary E. Landreth

**Affiliations:** 1 Department of Neurosciences, Case Western Reserve University, School of Medicine, 10900 Euclid Avenue, Cleveland, OH 44106 USA; 2 Stark Neurosciences Research Institute, Indiana University School of Medicine, 320 W 15th Street, Indianapolis, IN 46202 USA

**Keywords:** Microglia, Inflammation, Genetics, Genetic risk factors, Neurodegeneration, Alzheimer’s disease, Parkinson’s disease, Frontotemporal dementia, Triggering receptor expressed on myeloid cells 2

## Abstract

TREM2 variants have been identified as risk factors for Alzheimer’s disease (AD) and other neurodegenerative diseases (NDDs). Because TREM2 encodes a receptor exclusively expressed on immune cells, identification of these variants conclusively demonstrates that the immune response can play an active role in the pathogenesis of NDDs. These TREM2 variants also confer the highest risk for developing Alzheimer’s disease of any risk factor identified in nearly two decades, suggesting that understanding more about TREM2 function could provide key insights into NDD pathology and provide avenues for novel immune-related NDD biomarkers and therapeutics. The expression, signaling and function of TREM2 in NDDs have been extensively investigated in an effort to understand the role of immune function in disease pathogenesis and progression. We provide a comprehensive review of our current understanding of TREM2 biology, including new insights into the regulation of TREM2 expression, and TREM2 signaling and function across NDDs. While many open questions remain, the current body of literature provides clarity on several issues. While it is still often cited that TREM2 expression is decreased by pro-inflammatory stimuli, it is now clear that this is true in vitro, but inflammatory stimuli in vivo almost universally increase TREM2 expression. Likewise, while TREM2 function is classically described as promoting an anti-inflammatory phenotype, more than half of published studies demonstrate a pro-inflammatory role for TREM2, suggesting that its role in inflammation is much more complex. Finally, these components of TREM2 biology are applied to a discussion of how TREM2 impacts NDD pathologies and the latest assessment of how these findings might be applied to immune-directed clinical biomarkers and therapeutics.

## Background

Human genetic studies have provided crucial insight into neurodegenerative disease (NDD) pathogenesis. Alzheimer’s disease (AD) is a prime example of how advances in genetic technology have facilitated the evolution of our understanding of the etiology of NDDs. Early studies using genetic linkage approaches identified familial mutations in proteins related to amyloid beta production, amyloid precursor protein (*APP)*, presenilin 1 (*PSEN1)* and *PSEN2*, as well as the late onset AD (LOAD) risk variant apolipoprotein E4 (*APOE4*) [1]. These studies provided important insight into amyloid as a critical factor in AD pathogenesis and prompted application of molecular approaches and animal models to understand the disease. Since 2007 [2], case-control genome wide association studies (GWAS) have identified many novel AD-associated genetic variants [3]. Though many of these individually confer only modestly elevated risk for developing AD, collectively these studies provide broad insight into the pathways and processes involved in LOAD. Many identified genetic linkages are implicated in modulating immune function [4], demonstrating an important role for the immune response in AD.

More recently, next generation sequencing technologies have made possible the identification of rare variants, some of which may confer higher disease risk and therefore can provide important insight into genes with strong biological roles in disease [1]. The application of whole exome sequencing [5] and GWAS with imputation based on predicted genetic associations [6] to AD led to the identification of relatively rare variants in the gene triggering receptor expressed on myeloid cells 2 (*TREM2*) that are associated with a high risk for developing AD. Heterozygous *TREM2* variants confer similar risk for AD as one copy of *APOE4*. Significantly, the AD-associated *TREM2* variants are largely coding variants, in contrast to most of the single nucleotide polymorphisms (SNPs) identified in GWAS [7], making it more straightforward to translate into in vitro and in vivo models and perhaps also into therapeutics [8]. *TREM2* variants have now also been linked to other NDDs, suggesting that TREM2 is critically involved in shared disease mechanisms.

The excitement in the field following identification of these AD-associated TREM2 variants was also driven by its implications, providing a clear link between the innate immune system and NDD pathogenesis. While it has long been known that immune cell function is dysregulated in AD and other NDDs, it was not clear whether this actively contributed to disease pathogenesis and progression or was just a secondary response to AD-related pathology. However, this debate was largely settled in favor of the former when TREM2 variants were found to be significantly associated with risk for AD and other NDDs, and to form a genetic basis of polycystic lipomembraneous osteodysplasia with sclerosing leukoencephalopathy (PLOSL, also known as Nasu-Hakola disease). Because TREM2 is exclusively expressed on immune cells, these genetic associations were hailed as providing conclusive evidence that immune dysregulation can be a primary, causal contributor to NDD pathogenesis [9, 10]. Thus, NDD-associated TREM2 variants provide a new avenue to investigate the important roles that the immune system plays in neurodegeneration [11].

In the 4 years since TREM2 variants associated with AD risk were identified, many groups have developed research programs aimed at understanding TREM2 genetics, expression, structure, signaling, function, and its relationship to NDD pathologies and applied these findings to clinical biomarkers and therapeutics. Progress in these areas has clarified our understanding of the biology of the TREM2 receptor. While it was previously thought that TREM2 expression was decreased by pro-inflammatory stimuli and mediated anti-inflammatory effects, it is now clear that its roles are more complex. In vitro, inflammatory stimuli decrease TREM2 expression but in vivo TREM2 expression is increased in inflammatory contexts. More than half of studies report that TREM2 has a pro-inflammatory effect, suggesting that there must be cell type- and context-dependent functions of the receptor. Recent studies have also illuminated new aspects of TREM2 biology which necessitate a reevaluation and reinterpretation of previous literature. One example is the finding that soluble TREM2 is produced in AD in a disease progression-dependent manner [12] and that this soluble form of the receptor may have distinct biological effects [13, 14]. Other fundamental aspects of TREM2 biology are also under intense investigation, including epigenetic and posttranslational modification of TREM2 that affect expression and function, the ontogeny of TREM2 expressing cells in the brain, and how non-canonical signaling pathways may contribute to TREM2 function. This review offers a comprehensive synthesis of these studies alongside previous TREM2 literature to identify areas of consensus and emerging questions in the field. This understanding will be crucial to support informed design and interpretation of studies of TREM2 and the immune response in NDDs moving forward.

## Genetics of TREM2 in NDDs

### Diverse TREM2 variants are associated with NDD risk

There is great diversity in the TREM2 variants that have been associated with NDDs, including single amino acid substitutions, frameshift and nonsense mutations, and changes in splice sites predicted to alter the inclusion or exclusion of particular exons [15]. And, while most of the TREM2 variants identified are present in the coding sequence, there have also been disease-associated variants found in the 3’UTR [16], and upstream of the transcription start site [17]. The first NDD-associated TREM2 variants identified were W78X and W44X, which result in premature truncation of the protein, a variant at the consensus splice site which results in exclusion of exon 3, and the K186N mutation, which disrupts association of TREM2 with its obligate intracellular signaling adaptor, DAP12 [18]. These all likely result in a loss of TREM2 function, and a patient with the E14X nonsense variant of TREM2 had no detectable TREM2 transcript levels [19]. Subsequently, a host of diverse TREM2 variants have been identified. Despite their structural diversity, all of the NDD-associated TREM2 variants identified have been suggested to confer loss of function through different mechanisms. However, whether loss of function truly unifies all of these variants is still very much an open question.

### TREM2 variants are the genetic basis of PLOSL and some familial FTD cases

TREM2 was first identified as a genetic cause of PLOSL, also commonly known as Nasu-Hakola disease [20, 21], which is characterized clinically by bone cysts and fractures, neuropsychiatric symptoms and dementia [22]. Neuropathologically, PLOSL patients have axonal degeneration and white matter loss, as well as cortical atrophy [23, 24]. This is accompanied by an inflammatory response consisting of increased microglial density and activation and astrocytosis [25]. These neurological manifestations can also occur in the absence of fractures [26] or bone cysts [27]. Paloneva and colleagues [18] were the first to link TREM2 variants with PLOSL, and since then, many studies have identified homozygous TREM2 variants that form the genetic basis of PLOSL [28–33].

**Fig. 1:**
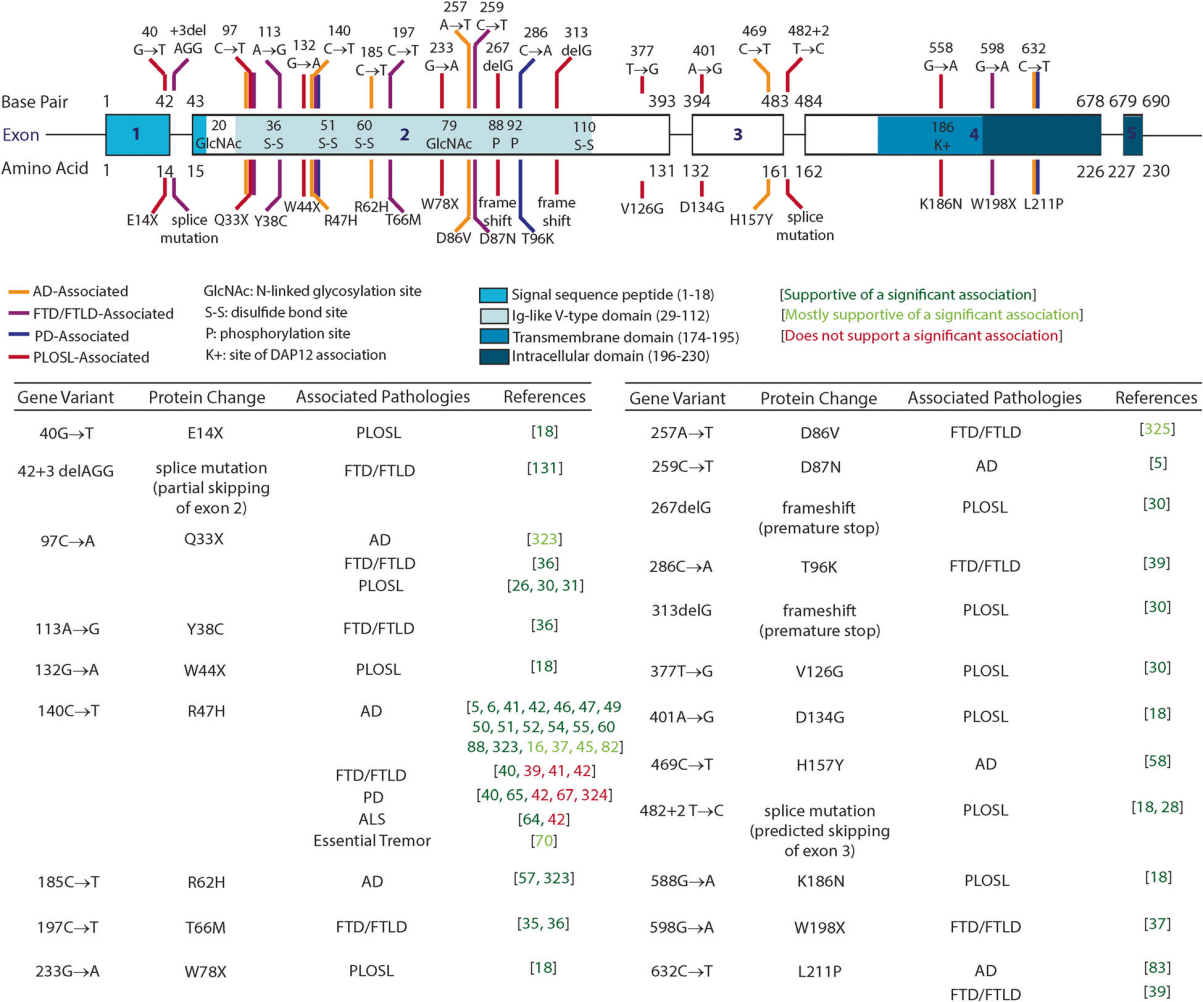
Diverse TREM2 variants are associated with NDDs. Genetic variants in the TREM2 gene (shown above) result in diverse changes in the protein structure (shown below). These variants occur in almost every exon (*black boxes*) and impact known protein motifs (sequences *highlighted in blue*) and flank many sites of known protein modifications (amino acid number and type of modification detailed inside *black boxes*). TREM2 variants have been found to be significantly associated with many NDDs, including AD (variants shown in *yellow*), FTD or FTLD (*pink*), PD (*purple*) and PLOSL (*red*). The table shows genetic variants that have been found to be significantly associated with disease risk, with supporting references shown in *dark green* and references that provide strong counterevidence shown in *red*. References shown in *light green* did find a significant association between the TREM2 variant and disease risk, but only in one or multiple populations they examined or only after inclusion of previously published literature into metastudy analyses. While these variants have been significantly associated with disease risk, many more studies find suggestive but not significant associations between additional TREM2 variants and NDD risk which are not represented here [5, 6, 16, 18, 26, 28, 30, 31, 35–37, 39–42, 45–47, 49–52, 54, 55, 57, 58, 60, 64, 65, 67, 70, 82, 83, 88, 131, 323–325]

Studies in families with frontotemporal dementia (FTD) [34] or frontotemporal lobar dementia (FTLD) found that PLOSL-associated TREM2 variants T66 M [35, 36], W198X [37], Q33X and Y38C [36] in either homozygosity or heterozygosity could also cause FTD [35–37] (Fig. 1). Case-control studies were then performed to assess whether TREM2 variants might increase risk for FTD in the general population. Initial studies suggested that there was a positive association between TREM2 variants as a whole and risk of FTD [16, 38] with a significant association found between FTD risk and individual TREM2 variants including T96K and L211P [39] and R47H [40]. Others failed to replicate this association with TREM2 variants and FTD or FTLD [34, 39, 41, 42]. However, an association between TREM2 variants and specific endophenotypes of FTD, including reduced white matter volume, seizures and motor symptoms has been reported [43]. Together, it is not clear whether TREM2 variants increase risk for FTD outside of specific familial cases, but they may influence specific clinical manifestations of the disease.

### TREM2 variants are associated with risk for AD

It was investigated whether TREM2 variants could also confer risk for Alzheimer’s disease. While it was first suggested that PLOSL-associated genes might confer risk for AD in 1983 [44], a small, case-control study in 2007 failed to demonstrate a significant association with AD risk [29]. However, larger studies in 2013 found that heterozygous expression of the TREM2 R47H [5, 6] and D87N variants [5] were significantly associated with AD risk. The association of TREM2 variants with AD has been extensively replicated [15, 16, 45–48] and the R47H variant [16, 37, 41, 42, 49–56] validated in neuropathologically-confirmed cases [57]. Other variants have also been consistently shown to confer AD risk, including D87N [5, 15], R62H [51, 56], L211P and T96K, and H157Y [51, 58] (Fig. 1). While rare, individuals with AD homozygous for the R47H [41, 50, 59] and L211P [41] variants have also been identified.

Studies examining the association of TREM2 variants with particular AD endophenotypes yielded somewhat inconsistent results, likely due to the small sample size of patients with these TREM2 variants. Some found that R47H TREM2 variant carriers had a decreased [41] or a trend toward decrease [54] in the age of AD onset, though others found no significant association [49, 60]. Additionally, some found that disease progression was accelerated in R47H carriers [60, 61], though this was not observed in all studies [41]. Other variants were also found to increase [62] or decrease [17] the rate of AD progression. Despite possible differences in AD onset or progression, the clinical presentation of AD in R47H carriers is similar to non-carriers [41, 49], though there may be a higher incidence of some endophenotypes including seizures and motor symptoms [63].

### TREM2 variants have been investigated as risk factors for other NDDs

TREM2 variants have also been assessed as potential risk factors for other neurodegenerative diseases, though the findings in these other disease contexts are less definitive. In amyotrophic lateral sclerosis (ALS), one study found a significant association of the R47H variant and ALS risk, as well as an inverse correlation between TREM2 levels in the spinal cord and survival in ALS patients [64], though this was not replicated by others [40]. The TREM2 R47H variant has also been reported to be associated with increased risk of Parkinson’s disease [39] by some [40, 65, 66] but not all studies [6, 42, 67]. In order to understand these differences, Lill and colleagues [42] divided their groups by ethnicity and found that the odds ratio of the R47H variant was significantly higher in their Northern European population compared to non-Northern Europeans. Others identified another Parkinson’s disease (PD)-associated SNP 5 kb upstream of TREM2 [66], though its effect on TREM2 expression is not known. The TREM2 R47H variant has also been investigated as a risk factor for posterior cortical atrophy [68], multiple system atrophy [69], essential tremor [70], multiple sclerosis [71] and Creutzfeldt-Jakob disease (CJD) [41], though these studies were not conclusive. One family has been identified in which a mutation in TREM2 is thought to result in progressive non-fluent aphasia [72], though other cases will be necessary to confirm this association. So far, evidence suggests that TREM2 variants are not significantly associated with dementia with Lewy Bodies [73], ischemic stroke [40] or progressive supranuclear palsy [40]. Because many of these diseases share overlapping clinical features with AD and FTD, it will be important to validate any associations of TREM2 with other NDDs in neuropathologically confirmed cases. Overall, the association of TREM2 variants with these other NDDs is less clear, and future studies with large sample sizes in diverse but well-matched populations will be required to definitively establish whether TREM2 variants confer risk for NDDs other than PLOSL, FTD and AD. Importantly, the association of TREM2 variants with multiple NDDs suggests it may underlie common disease mechanisms. TREM2 dysfunction may provide insight into mechanistic links among these diseases.

### The epidemiology of TREM2 variants

Epidemiologically, the prevalence of TREM2 variants differs greatly among individuals from different genetic backgrounds [74]. In Caucasian populations, the minor allele frequency (MAF) of the R47H TREM2 variant ranges from 0.12–0.26% in the United States, to up to 2% in some specific British populations [5, 6, 59]. While the R47H variant is virtually absent in East Asian individuals, [56, 69, 75–81] nine other TREM2 variants were present in East Asian populations and collectively associated with NDD risk [82]. Similarly, the MAF of the R47H and R62H variants are much lower in African Americans compared to European American populations [83, 84]. However, exonic sequencing of TREM2 revealed variants that had much higher MAFs in the African American population compared to European Americans, and some of these variants were significantly associated with AD risk in that population [83]. Because of the small MAFs, it is not clear whether the effect sizes of these variants differ among different ethnic groups as well. The low MAFs and diversity in the frequency of TREM2 variants across populations necessitates that studies have large study populations and be well-matched for ethnicity. It will also be important to take advantage of the identification of these TREM2 variants across diverse populations to gain a full understanding of how TREM2 variants confer AD risk and how they might interact with other genetic differences among individuals from distinct genetic backgrounds.

### The relationship between TREM2 and other NDD genetic risk factors

Several groups have examined the relationship between TREM2 and other NDD risk factors. Variants in Siglec-3 (CD33) are significantly associated with AD risk and CD33 levels on human blood monocytes were found to inversely correlate with surface TREM2 levels [85]. The AD-associated CD33 allele increased its surface expression, effectively decreasing TREM2 signaling. Another AD-associated gene, Membrane-spanning 4-domain family A (MS4A), was found to be co-enriched with TREM2 in the human brain and is substantially upregulated in monocytes derived from patients with PLOSL-associated TREM2 variants [86]. An AD-associated SNP in MS4A correlated with altered soluble TREM2 (sTREM2) levels in cerebrospinal fluid (CSF), suggesting that MS4A may also regulate TREM2 expression or processing [86]. TREM2 levels are also increased in AD mouse models lacking PGRN expression, which models lower PGRN expression observed with an AD and FTLD-associated PGRN genetic variant [87].

Components of the TREM2 signaling pathway have also been associated with NDDs, including its intracellular signaling adaptor DNAX activation protein of 12 kDa (DAP12, also termed TYROBP). DAP12 and TREM2 variants produce virtually indistinguishable phenotypes in PLOSL patients [18, 30]. However, Satoh and colleagues [25] found that dendritic cells derived from monocytes of PLOSL patients with TREM2 or DAP12 mutations had very different patterns of gene expression, suggesting that they may produce the same phenotype through different molecular mechanisms. A rare DAP12 variant at the site of interaction with TREM2 also confers risk for developing early onset AD [88] and DAP12 was found to play a central role in AD-related molecular networks [89]. As its relationship to NDDs might predict, DAP12 deficient mice also have synaptic degeneration and reduced myelination [90], though recent evidence suggests that DAP12 deficiency may be neuroprotective in an AD mouse model [91]. The precise mechanisms underlying these changes are not yet understood. Variants in additional proteins associated with the TREM2 signaling pathway, SHIP1 and colony stimulating factor 1 receptor (CSF1R), have been associated with AD risk and leukoencephalopathy with spheroids [92, 93]. In addition, ApoE, a putative TREM2 ligand [94–96], is clearly established as an AD risk factor [97]. Finally, environmental risk factors for AD including traumatic brain injury [98, 99], diabetes [100] and age [101], all alter TREM2 expression in the brain. Together, the identification of variants in genes involved in these common immune pathways suggest that TREM2, along with its interaction partners, together play an important role in modifying NDD pathology.

### TREM2 expression

#### Co-regulation of TREM2 and other members of the TREM family

TREM2 is located on human chromosome 6 in a gene locus containing several TREM and TREM-like genes, such as TREM1, triggering receptor expressed on myeloid cells like transcript 1 (TREML1) and TREML2, which likely originated from duplication events but now have relatively diverse sequences [102–104]. Many of these genes, including TREM2, are highly conserved between humans and mice, while others are present only in mice (TREM3 and TREML6) or humans (TREML3 and Nkp44). There may be some shared mechanisms of gene regulation across the locus. For example, there is a Retinoid X receptor (RXR) binding site upstream of the entire locus that is thought to result in coordinate regulation of these genes [105]. However, in some cases, opposing regulation of these different genes has been shown, such as between TREM2 and TREM1 [85], and between TREM2 and TREML2 [106, 107]. It is not known what factors contribute to these inverse correlations in expression. SNPs within this locus can also result in changes in expression of multiple TREM genes. Variants in the TREML2 [66] and TREML4 [108] gene have been shown to increase brain TREM2 and TREML1 expression levels. Chan and colleagues [85] found that AD-associated variants in TREM1 result in reduced TREM1 expression on human monocytes and increased TREM2 expression. Moreover, an intronic variant in TREM1 which decreases its expression leads to increased amyloid accumulation and cognitive decline in AD patients [109]. However, non-AD associated variants decreased both TREM1 and TREM2 expression, leading the authors to suggest that the ratio of TREM1 and TREM2 expression rather than the absolute changes in expression may be important for disease. Additionally, variants in TREML2 increase PD risk [62], and other disease-related SNPs that alter TREML2 levels [66] associate with AD in GWAS analyses [93]. Another variant of TREML2 was found to be protective against developing AD [107]. An intergenic variant associated with AD risk was shown to alter RNA levels of TREML4 [110]. These data highlight the importance of characterizing the expression patterns of all TREM members in an effort to pinpoint co-regulatory mechanisms among these genes physiologically and in the context of disease.

#### Regulation of TREM2 expression

TREM2 expression is highly cell-type and context specific. However, the molecular mechanisms governing this highly specific regulation of TREM2 expression are just beginning to be understood. While there are several predicted transcription factor binding sites in the UTR’s and promoter region of TREM2, only a few have been functionally validated. PU.1, a master regulator of myeloid cell fate specification, is present in the TREM2 promoter [111]. The RXR agonist bexarotene was found to enhance RXR occupancy of known binding sites [105] upstream of TREM2 in mice, though this did not correlate with increased TREM2 RNA levels [112]. However, in AD mouse models, bexarotene did enhance gene expression of TREM2 [112], suggesting that RXR may effectively induce TREM2 transcription when cells are already primed in a particular context. NKκB [113, 114], Protein E [115], RANKL, and NFAT [116] have all been shown to regulate TREM2 expression in different cell types, but it is not clear whether these factors directly regulate expression. One group has suggested that NFκB may instead regulate TREM2 expression by increasing levels of microRNA34a which, in reporter assays, decreased TREM2 expression [113, 117–121]. There are also epigenetic changes that have been shown to influence TREM2 expression. In humans, hippocampal enrichment of 5-hydroxymethylcytosine (5hmc), a marker of active demethylation, at the TREM2 transcription start site and in exon 2 were found to positively correlate with TREM2 mRNA levels [122]. Methylation upstream of the TREM2 transcription start site was also increased in AD, a context in which TREM2 levels are also increased [123]. Additionally, methylation at the CpG sites in intron 1 negatively correlated with TREM2 mRNA levels in human leukocytes, and methylation at these sites was reduced in AD patients [124]. Others have found that H3Kme2 and H3Kme3, histone modifications which typically are associated with active gene transcription, are increased at the TREM2 locus in db/db mouse adipose tissue [125] and in cultured dendritic cells and macrophages during differentiation [126], contexts in which TREM2 mRNA levels are elevated.

Finally, there are likely post-transcriptional mechanisms of regulation of TREM2 expression which contribute to differential expression in different contexts. TREM2 mRNA stability can be dynamically regulated. The half-life of TREM2 mRNA in cultured human peripheral blood mononuclear cells went from 11.3 h to 4 h after toll-like receptor (TLR) ligation [127]. Furthermore, Hu and colleagues [128] found that TREM2 mRNA expression strongly correlated with surface levels of TREM2 protein on monocytes, but only weakly correlated with protein expression on granulocytes in the plasma. This suggests that, in addition to differential regulation of TREM2 transcription through transcription factors and epigenetic markers, TREM2 expression can be further differentially controlled at the mRNA and protein level in distinct cell types through mechanisms which are not resolved.

How TREM2 variants affect TREM2 expression is a topic currently under investigation. As discussed above, some TREM2 variants are known to reduce TREM2 expression. The nonsense mutations E14X [18] and Q33X [129] were both found to eliminate TREM2 protein expression. TREM2 RNA levels were reduced in patients harboring the variant T66M [23] and a splice donor mutation in intron 3 [130]. Heterozygous expression of a variant that affects TREM2 splicing in intron 1, which is associated with early onset dementia, also affected the expression of the common variant allele of TREM2, reducing it by more than half [131]. It is unclear how these variants result in reduced TREM2 transcript levels, though it has been suggested that epigenetic changes may partially account for these effects. In contrast, the R47H variant either did not change [57] or trended toward increasing TREM2 transcript levels [132] in individuals with AD. Although there were no changes found in gene methylation upstream of TREM2’s transcription start site in R47H carriers compared to controls [123], variants tend to cluster around exon 2 where TREM2 expression was found to be correlated with 5hmc enrichment [122]. Whether variants affect regulation of TREM2 RNA expression at the level of epigenetics, transcription factor binding, RNA stability or altering the cell phenotype in a manner that indirectly drives alterations in TREM2 transcription is not yet clear. At the protein level, in transfected cell lines T66M and Y38C TREM2 variant proteins were found to be degraded by the proteasome, leading to decreased protein expression [133]. In AD patients with R47H variants, there was a trend toward decreased TREM2 protein levels in one study [57], but a trend toward an increase in another [132]. It will be crucial to continue collecting data from TREM2 variant carriers to gain a clearer picture of how TREM2 expression is altered by different disease-associated variants. In PLOSL patients with DAP12 mutations, there was a variable effect on DAP12 expression levels, in some cases increasing and in others decreasing expression [23]. It may be that TREM2 variants also alter TREM2 expression in distinct ways.

#### Cell types in which TREM2 is expressed

**Fig. 2:**
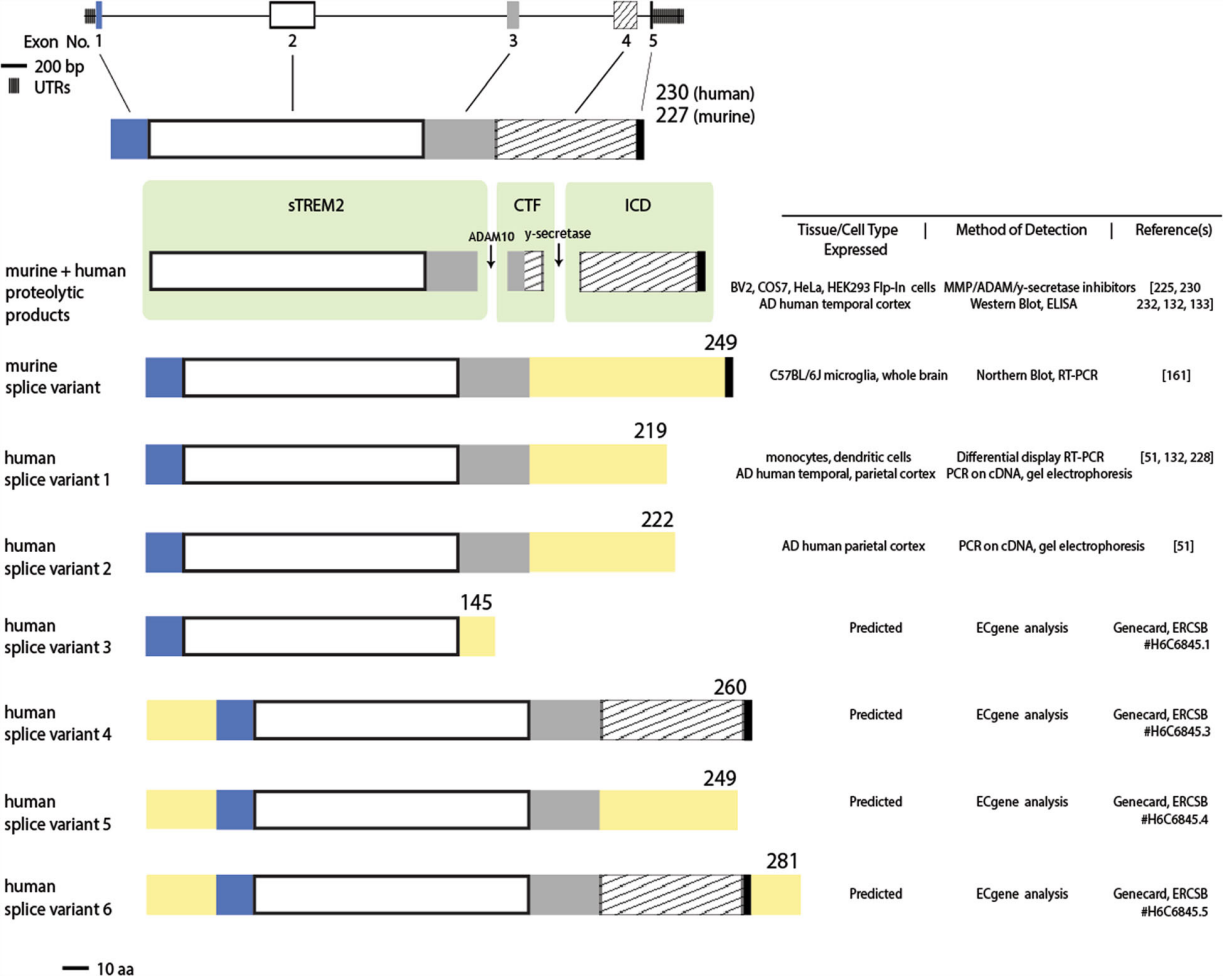
TREM2 can be present as a full-length protein (shown at *top*) or as a soluble product. This can occur through proteolytic cleavage by ADAM10 followed by γ-secretase to produce soluble TREM2 (sTREM2), a C-terminal fragment (CTF) and an intracellular domain (ICD). TREM2 can also be alternatively spliced to produce soluble isoforms. One alternative transcript has been validated in mice, while two have been validated in humans and four others are predicted to occur [51, 132, 133, 161, 225, 228, 230, 232]

TREM2 is expressed on many cells of the myeloid lineage, as its name suggests, including dendritic cells [101, 126, 130, 134–136], granulocytes [128], bone marrow and monocyte derived macrophages [126, 130, 135, 137, 138], and tissue macrophages like splenocytes [139], Kuppfer cells [140], alveolar macrophages [141, 142], and osteoclasts [19, 116, 143] (Fig. 2). TREM2 is not reported to be expressed on lymphocytes [128]. Its expression on circulating monocytes remains controversial. Initially it was thought that TREM2 was only expressed after differentiation of monocytes into macrophages [130], and others have provided further data to support a lack of TREM2 expression or expression on only a small subset of monocytes in humans [101, 126, 135, 144] and mice [145]. However, others have detected TREM2 expression in whole blood [124, 146–148] and specifically on human [85, 128] and mouse [149] monocytes. These disparate findings may be due to differences in sensitivity of detection or other technical factors, but it will be important to resolve moving forward.

It is clear that TREM2 is expressed in the brain and most evidence documents that TREM2 is expressed exclusively within the brain by microglia. All published studies to date find that primary cultured microglia express TREM2 [139, 150–153]. In vivo*,* most studies detect TREM2 expression in mouse microglia [11, 101, 152–157], though some find that it is expressed by only a subset of these cells [114, 158–161] and others could detect TREM2 RNA but not protein expression in mouse microglia [162]. Furthermore, studies demonstrated that when microglia are acutely depleted from the brain through CSF1R antagonist treatment in vivo*,* [163] through CD11b-HSVTK depletion in brain slices [155], or chronically in PU.1 knockout mice [154], TREM2 is no longer detectable in the CNS. In humans, experimental outcomes have been more variable, with some detecting high levels of TREM2 expression across all microglia [156], others finding lower levels of TREM2 expression [164] and others not detecting microglial expression of TREM2 [25]. With a few exceptions, most evidence is in strong agreement that TREM2 is expressed, at least under normal physiological conditions, specifically in microglia within the CNS.

#### TREM2 expression changes throughout neurodevelopment and varies across brain regions

TREM2 expression in the CNS is also regulated throughout development and its expression pattern varies across different brain regions. TREM2 is first detectable in the mouse CNS at E14 and continues to be expressed through adulthood [154]. However, at P1, all brain myeloid cells express TREM2 RNA, but not all of these cells still express detectable levels by P10, which has also been supported in vitro [165]. This early elevated TREM2 expression also occurs in other organs following macrophage infiltration during organogenesis [137]. TREM2 expression is also regulated differentially across brain regions in humans and mice. TREM2 was found to be highly expressed in white matter [101, 165], hippocampus [101, 156, 165] and spinal cord [18, 156], among other regions. While this could suggest that particular microenvironmental niches induce TREM2 expression locally within distinct microglial populations, it may also simply reflect the high density of microglia in these regions [101].

#### Inflammatory stimuli, injury and disease drive changes in TREM2 expression

TREM2 expression has been shown to be dramatically altered in the contexts of inflammation, injury and disease. In vitro, application of classically pro-inflammatory molecules (TNFα [117, 136], IL1β [107, 136, 166], ROS [166, 167], IFNγ [168], TLR agonists, including lipopolysaccharide (LPS) [106, 114, 135, 136, 167–170], CpGs [171] and other TLR ligands [114, 127, 138, 150], mitochondrial lysates [172] and bacteria [173]) decreased TREM2 expression, while anti-inflammatory molecules (vasoactive intestinal peptide [174] and IL4 [168]) increased TREM2 expression. In contrast, in vivo*,* inflammation and different disease states almost universally increase TREM2 expression. Stimuli that induce an inflammatory response in the lung increase TREM2 expression in alveolar macrophages [141, 149, 173–178]. High fat diet [100, 179] increased inflammation and TREM2 expression in adipose tissue, liver and brain. TREM2 is also upregulated in numerous other inflammation-related contexts, including sepsis [180], rheumatoid arthritis [181], corneal infection [182], macular degeneration [117], glioma [183], oral [184], esophaegeal [185], and liver [186] cancers, following prosthetic joint implants [187], osteoporosis [188], colonic mucosal injury [189], colitis [190], gastrointestinal mucositis [191] and muscular sarcoidosis [192]. In the CNS, TREM2 expression is increased in the context of traumatic brain injury [98, 99], stroke [160, 193], spinal nerve transection [194], ALS [64], PD [66], prion disease [155, 195], models of demyelination [151, 159, 196–198] and following beta-amyloid (Aβ) vaccination [199]. Only one study found reduced TREM2 expression in in vivo inflammatory contexts, following LPS injection and middle cerebral artery occlusion in mice [114]. TREM2 has also been shown in almost all cases to positively correlate with aging, both in mouse models [200, 201] and in humans [101]. Soluble TREM2, a product of full-length protein cleavage or alternative splicing, is detectable in CSF and its levels were also positively correlated with age [12, 86, 202, 203], though this was not reflected in blood [204]. Taken together, these studies largely demonstrate that, in vitro*,* inflammatory stimuli decrease TREM2 expression, while in vivo*,* inflammatory stimuli predominantly increase TREM2 expression, clearly suggesting that the dogma based on early studies that TREM2 expression is universally reduced in inflammatory contexts is not applicable to in vivo contexts. Why this occurs is not clear, but may reflect differences in cell recruitment, acute versus chronic signaling, non-cell autonomous signaling pathways, or phenotypic changes in myeloid cells that occur when they leave their native environment.

The most comprehensive assessment of changes in TREM2 expression has been performed in the context of Alzheimer’s disease. Studies in AD patient brain tissue almost exclusively show increased TREM2 expression [122, 132, 150, 164, 205–208], and some [124, 128, 148] but not all [146] found this was also reflected in increased TREM2 levels in monocytes from AD patients. TREM2 levels in the brains of AD mouse models are also increased. One study reports a reduction in TREM2 RNA levels before the onset of pathology in Tg2576 mice [209], though after the onset of pathology, all amyloid models of AD examined have increased TREM2 RNA and protein levels [162, 200, 210–214]. This upregulation of TREM2 expression occurs shortly after the onset of pathology and largely seems to correlate with amyloid burden [85, 157, 215] and the association of myeloid cells with amyloid plaques [207, 208]. Tau models of AD also show increased TREM2 levels [211, 216], however, TREM2 is only increased long after neurofibrillary tangle development in these models [211], consistent with TREM2 upregulation at late stages of disease progression in postmortem AD brain tissue [207]. Several studies have sought to determine what aspect of AD pathology drives TREM2 expression. TREM2 is upregulated in myeloid cells associated with plaques [158, 162, 212, 213, 217, 218], and specifically, TREM2 is highly expressed on myeloid cell processes in contact with plaques [219]. In support of a plaque-driven upregulation in TREM2 expression, Varvel and colleagues [217] depleted microglia from the brains of an AD mouse model and allowed the brain to repopulate with new myeloid cells. These new cells that repopulated the brain initially failed to associate with plaques, but eventually became plaque-associated, and coordinately upregulated TREM2 expression. Furthermore, stereotactic injection of beta-amyloid 42 (Aβ42) into the cortex and hippocampus of wild-type mice was sufficient to induce an upregulation in TREM2 transcripts within 24 h [214]. Together, these findings suggest that amyloid can increase TREM2 expression in myeloid cells. To determine whether this effect was cell-autonomous, cultured microglia were treated with Aβ, though these studies have so far produced inconsistent results with respect to TREM2 expression [106, 214]. Significantly, Melchior and colleagues [158] found that there was no effect on cultured microglia treated with beta-amyloid 40 (Aβ40) on TREM2 levels, but if they added Aβ40 to mixed glial cultures, this did result in upregulation of TREM2 on microglia by flow cytometry. This suggests that, at least this Aβ species may drive TREM2 expression through feedback from other cell types, though the signals that mediate Aβ-induced upregulation of TREM2 on myeloid cells are not yet known.

#### TREM2 expression by peripherally derived macrophages in the AD brain

Not all myeloid cells associated with plaques express TREM2 [158, 162]. Investigation of which subset of myeloid cells upregulated TREM2 in the AD brain has yielded conflicting results. Some findings suggest TREM2^+^ cells may be peripherally derived macrophages rather than brain resident microglia. Jay and colleagues [162] found that TREM2 was expressed on CD45^hi^ myeloid cells which expressed the monocyte marker Ly6C and not the microglial-specific marker P2RY12. Following *toxoplasma gondii* infection in 5XFAD mice, Mohle and colleagues [220] found that TREM2 was expressed most highly by C-C chemokine receptor type 2 (CCR2)^+^Ly6C^lo^F4/80^+^ cells in the brain, a marker signature of peripherally derived macrophages. TREM2 expression in other disease models, including basal cell carcinoma [138], sciatic nerve transection [221] and colonic mucosal injury [189], was found to be upregulated coincident with the peak of macrophage infiltration into the tissue. In contrast, others have detected TREM2 expression on “dark” microglial cells in the brain, which are 4D4^+^ and 4C12^−^, a marker signature consistent with resident microglia [215]. Wang and colleagues [222] also performed experiments in which age-matched CD45.1 WT donors were parabiosed with CD45.2 AD mouse models and were not able to detect CD45.1 cells in the brains of the AD mice. Indeed, the contribution of peripherally derived macrophages to NDD pathologies has a long and controversial history. However, if TREM2 is expressed on these cells, it suggests they may play a key role in modulating pathology, and thus this issue should continue to be explored.

## TREM2 structure and signaling

### The structure of full-length TREM2

TREM2 is a single pass transmembrane protein whose ligand binding domain includes an extracellular Ig-like domain [223] with N-linked glycosylation sites [133, 136, 170, 224], phosphorylation sites and disulfide bonds which are thought to perform important structural roles. The transmembrane domain anchors TREM2 to the membrane and contains the intramembraneous lysine residue necessary for association with its intracellular membrane adaptor, DAP12. This is followed by a short cytoplasmic tail with no established function. Disease-associated variants of TREM2 alter many of these structural elements (Fig. 1). Many variants are found in exon 2 and may change the structure of its ligand-binding domain, impacting the affinity of TREM2 for different ligands. For example, the Y38C variant associated with PLOSL and FTD is predicted to alter an important flanking sequence of the cysteine residues which form TREM2’s disulfide bonds [36, 223]. Likewise, glycosylation is affected in cells transfected with Y38C and T66 M TREM2 variants [225], though not in several other variants examined [48]. Glycosylation of the R47H variant was also found to be reduced [225], though not to the same extent as the other TREM2 variants in vitro. In humans expressing the R47H variant there were no significant differences in the level of glycosylation [132] but there were differences in the pattern of glycosylation [133, 224]. TREM2 variants can also affect other important structural motifs of the TREM2 protein such as those required for DAP12 association, and overall protein folding and stability. T66M and Y38C variants [223] along with V126G are predicted to be important for protein packing. Consistent with impaired protein folding, T66M and Y38C variants exhibit enhanced proteasomal degradation [129]. The R47H variant has been predicted to impair protein stability [226], but transfected R47H-TREM2 constructs actually have an increased half-life relative to WT TREM2 and are resistant to proteasomal degradation in the endoplasmic reticulum (ER) [224]. NDD-associated TREM2 variants located on the surface of the protein (R62H, T96K, D87N and R47H), are not predicted to substantially alter TREM2 structure [223, 227] but instead affect ligand binding [223]. As the structure of TREM2 and disease-associated variants continue to be resolved, we will gain better insight into the organization of structural features essential for TREM2 function.

### The structure and production of soluble TREM2

**Fig. 3:**
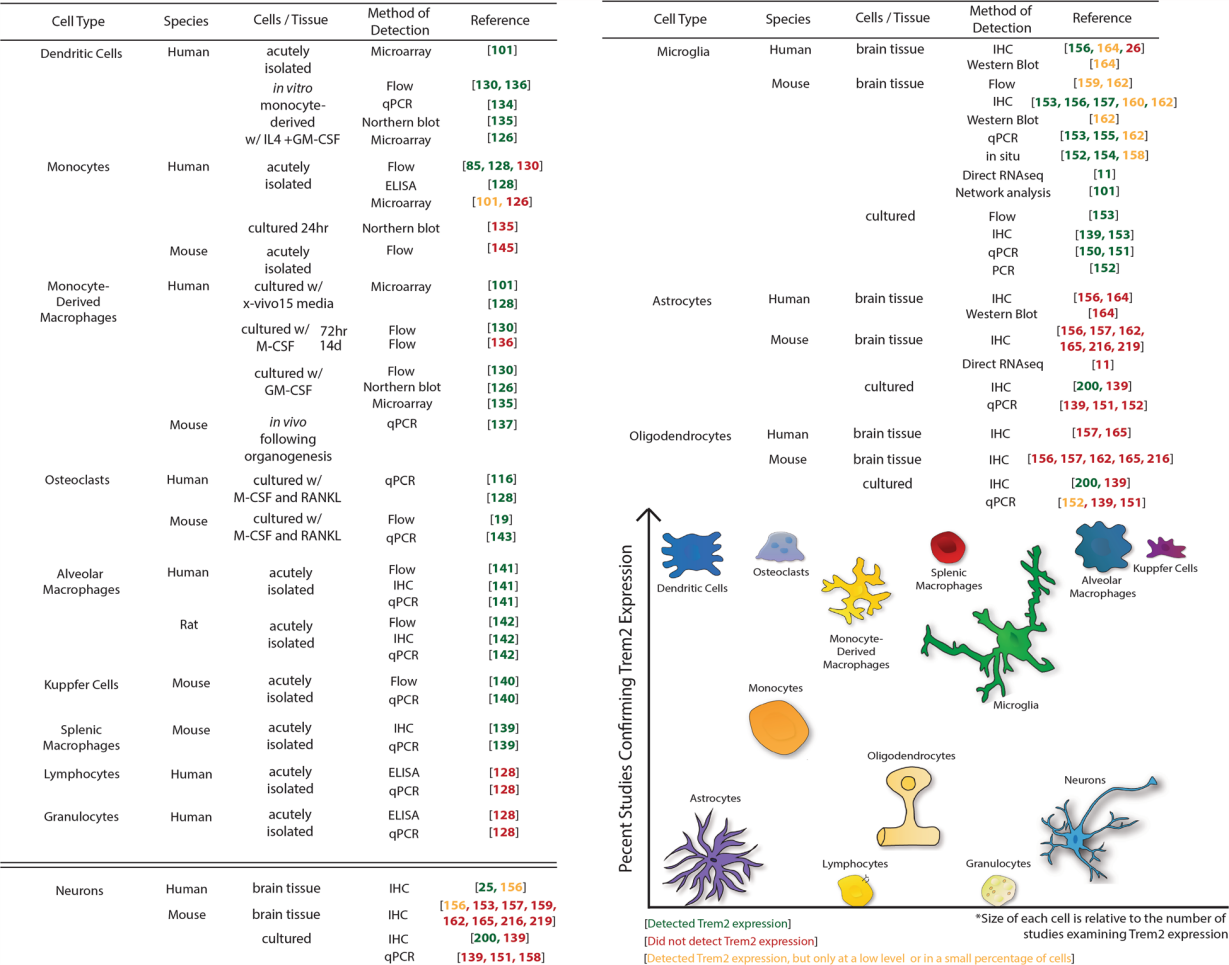
TREM2 is expressed in many immune cells, and is localized to microglia in the CNS. TREM2 expression has been assessed in a variety of human and mouse cell types. These data represent TREM2 expression in these cells under homeostatic conditions, though, as discussed in the next section, TREM2 expression can change in the contexts of inflammation or pathology. References shown in *green* are supportive of TREM2 expression in the cell type listed while those in red did not detect TREM2 expression using the listed method of detection. References in *yellow* provide evidence of expression, but at low levels or in a small percentage of cells assayed. The *graph* represents the cell types in which TREM2 expression has been examined at a size relative to the number of studies and methods used to detect TREM2 expression in that cell type. They are graphed along the y-axis according to the percentage of these findings which support TREM2 expression on these cells [11, 19, 25, 26, 85, 101, 116, 126, 128, 130, 134–137, 139–143, 145, 150–160, 162, 164, 165, 216, 219, 200]

TREM2 can also be produced as a soluble protein (Fig. 3). Soluble TREM2 (sTREM2) has been detected in the supernatants of mouse [225] and human cells in culture [198, 225]. It has been proposed that sTREM2 could be produced by both alternative splicing and proteolytic cleavage. Insertions [161] or frameshifts [228] preceding exon 4 terminate the transmembrane domain and are predicted to yield a soluble product. In human brain tissue, at least three TREM2 isoforms have been detected [51], with isoform 1, encoding the full-length protein, being the most highly expressed [122]. Meanwhile, the transcript encoding a 219-residue splice isoform is expressed to a lesser extent than isoform 1 in the hippocampus of AD patients [132], whereas the degree of expression of the 222-residue splice isoform has yet to be resolved. Based on RNA sequencing data in AD mice, 15–20% of transcripts were predicted to be alternatively spliced [208]. Notably, these alternative transcripts have been identified in human monocytes [228], and in AD brain tissue [51, 132]. Evidence of elevations in expression of TREM2 exons 3 and 4 in advanced AD cases by microarray-based gene expression analysis [229] is suggestive of TREM2 alternative splicing in AD [51, 132, 228]. The expression of full length and splice isoforms of TREM2 are strongly correlated in AD tissue, suggesting all TREM2 isoforms may be coordinately regulated [132]. DNA methylation within the body of the gene has been shown to impact alternative splicing [123], and as TREM2 can be methylated within exon 2 [122], it is possible that context-dependent changes in methylation may also result in altered splicing. However, direct evidence that alternatively spliced mRNAs are translated is lacking.

The sequential proteolytic processing of TREM2 has definitively been shown to generate sTREM2 [132, 225, 230]. In vitro inhibitor studies revealed full-length TREM2 is proteolytically cleaved by a disintegrin and metalloproteinase domain-containing protein (ADAM10) resulting in shedding of the ectodomain [136, 225, 230]. Detection of TREM2 C-terminal fragments (CTFs) within cell lines [133, 225, 230] and human brain extracts [231] is suggestive of a two-step proteolytic cleavage event of TREM2. Following ectodomain shedding by ADAM10, the remaining membrane-associated TREM2 C-terminus undergoes intramembrane proteolysis by γ-secretase to release its intracellular domain [230]. γ-secretase cleavage of TREM2 results in accumulation of TREM2 CTFs, without a corresponding increase in full-length TREM2 at the plasma membrane upon γ-secretase inhibition [133, 230, 232]. It is not known how this process is regulated, but it is known that TREM2’s adaptor protein DAP12 is required for sTREM2 production, at least in some contexts [149].

Whether the production of sTREM2 in AD occurs by alternative splicing or ectodomain shedding is unclear. It may be cell-type and context dependent as different cell types showed either an up- (dendritic cells) or downregulation (microglia, monocytes) of the TREM2 splice transcript levels when stimulated by LPS [161, 228]. IL13 and IL4 were also shown to enhance sTREM2 production in bone marrow derived macrophages [149]. This is consistent with studies examining other members of the TREM family as TREM1 [233–235] and TREM-like transcript-1 [236] which also produce cell-specific protein products.

TREM2 variants linked to AD and other neurodegenerative diseases can alter soluble TREM2 generation. TREM2 variants can drive novel TREM2 splicing [28, 32] such as the PLOSL-associated TREM2 variant c.482 + 2 T > C in which conversion of a single nucleotide at a splice-donor consensus site within intron 3 results in the deletion of exon 3 in addition to exon 2 and/or exon 4 [18]. This is proposed to produce soluble protein products [28] lacking either the transmembrane (TM) domain or both the TM and ectodomain. While most TREM2 variants are expressed in all isoforms of TREM2, there are disease-associated variants expressed only in the alternatively spliced isoforms of TREM2 [15, 16, 51, 58, 83]. Several reports have also demonstrated that TREM2 variants T66M and Y38C yield significant changes in sTREM2 release by cultured cells [86, 225]. This was also evident in individuals homozygous [203, 225] and heterozygous [86] for the T66M variant, exhibiting a loss or reduction in CSF levels of sTREM2, respectively. Other case reports showed disease-associated TREM2 variants lowered CSF sTREM2 levels compared to a non-carrier group [86]. While some have reported decreased levels of sTREM2 and TREM2 intracellular domain (ICD) production in vitro with the R47H variant [224], the TREM2 R47H variant acted similarly to wild-type in sTREM2 production in other in vitro assays [225] and AD patients carrying the R47H variant had elevated levels of CSF sTREM2 [86]. There was a trend for higher transcript levels of a 219-residue TREM2 splice variant in cortices of late-stage AD cases with the R47H risk allele compared to significant elevation in this splice isoform in non-carrier AD cases [132]. These data altogether suggest that different TREM2 variants have distinct effects on sTREM2 levels.

### Subcellular localization of TREM2

In addition to altering proteolytic cleavage, the localization of TREM2 within the cell can greatly impact TREM2 signaling, and its trafficking appears to be a highly dynamic process. In homeostatic conditions, TREM2 seems to be primarily found intracellularly [237], associating with the trans-Golgi network [156, 200, 238] and in a population of exocytic vesicles [238]. These vesicles appear to be continuously shuttled to the membrane, a process which can be rapidly induced by increases in Ca2+ in response to ionomycin [238]. It is not clear what other specific stimuli or disease contexts result in changes in TREM2 localization within the cell, but this will be critical to understand TREM2’s functional role in these contexts. TREM2 is recycled from the membrane in clatherin-coated vesicles in a beclin-1 [239] and Vps35-dependent manner [239, 240]. Vps35 mediates recycling of TREM2 from the membrane via retromer complexes [240]. When this process is blocked, TREM2 increases its association with the lysosome and is degraded [240]. TREM2 variants can impact TREM2 localization within the cell. In cells in which TREM2 variants were transfected, T66M and Y38C [48, 133, 223, 225, 237] as well as other variants [48] significantly reduced TREM2 surface expression. These variants increased the localization of TREM2 with the ER [133, 225], which may indicate impaired protein folding. The R47H variant was found to either not alter surface expression [48, 133] or reduce surface expression of TREM2 to a lesser extent [225]. Unlike the other variants, R47H TREM2 was mostly localized to the trans-Golgi network rather than the ER, comparable to the WT receptor [133, 225]. However, Yin and colleagues [240] did find that the R47H variant had reduced association with Vps35, resulting in increased lysosomal degradation following recycling of the receptor from the surface. Overall, this suggests that variants may, in part, impact TREM2 function by altering the localization of TREM2 within the cell. While TREM2 has been proposed to play a functional signaling role exclusively on the cell surface, this could also impact possible functional roles of TREM2 in other cellular compartments.

### TREM2 ligands

**Fig. 4:**
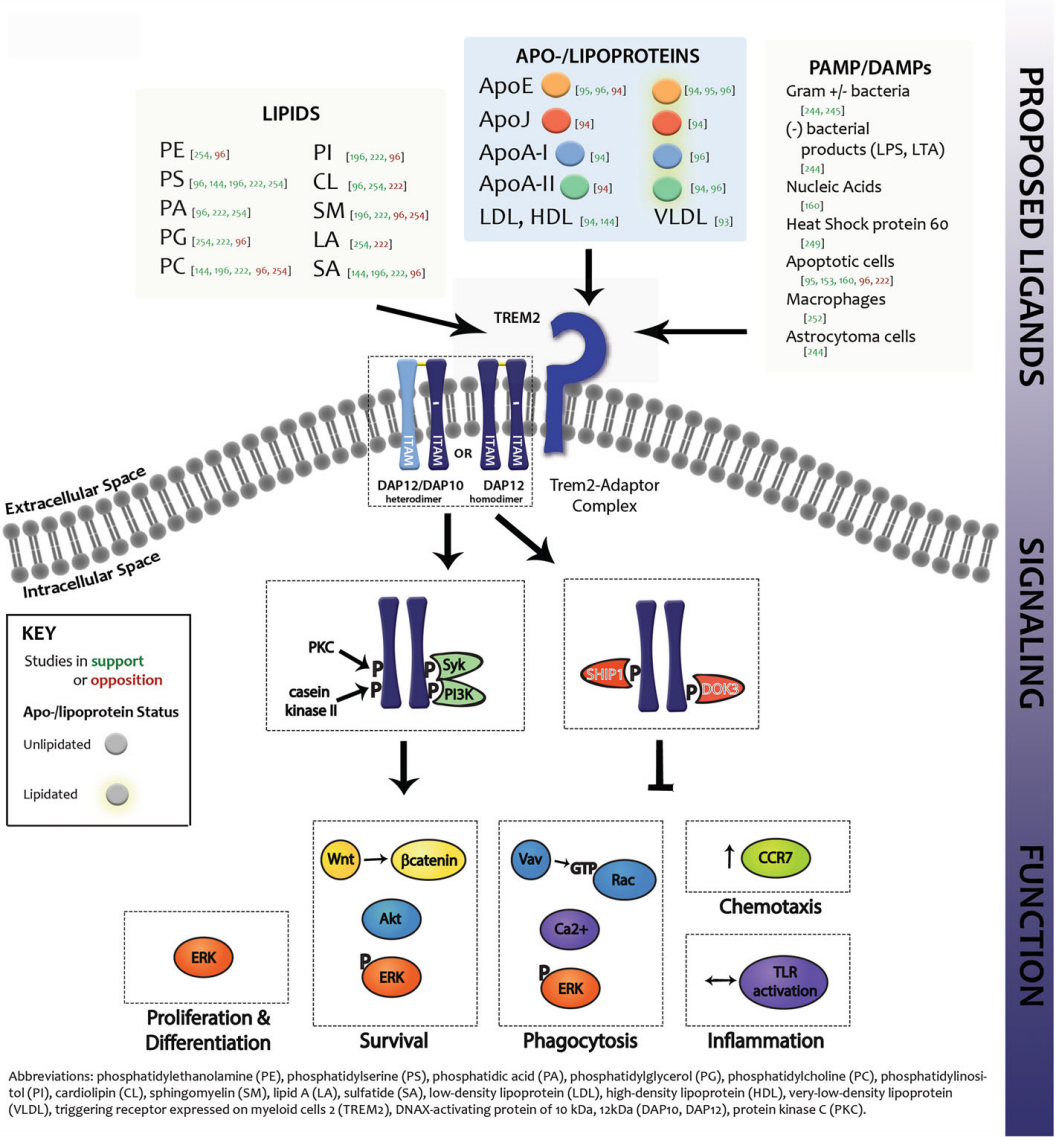
TREM2 signaling and function. TREM2 has been proposed to bind to a variety of different ligands, categorized here by lipids, lipoproteins and ligands associated with damage- or pathogen-related molecular patterns. These ligands bind to the TREM2 receptor. Following ligand binding, TREM2 can associate with DAP12 homodimers or DAP12/DAP10 heterodimers to mediate downstream signaling. This signaling requires phosphorylation of the adaptor, following which activating (shown on left in *green*) or inhibitory (shown on right in *red*) signaling components can bind. These activating components have been shown to initiate different downstream pathways that lead to cell proliferation and differentiation, survival, phagocytosis, chemotaxis and inflammation. While many other signaling components are thought to play a role downstream of TREM2 activation, only those validated as part of the signaling pathway responsible for the listed functions have been included here. Association with inhibitory components is thought to prevent activation of these downstream pathways. Lipids: PE [254, 96], PS [96, 144, 196, 222, 254], PA [96, 222, 254], PG [254, 222, 96], PC [144, 196, 222, 96, 254], PI [196, 222, 96], CL [96, 254, 222], SM [196, 222, 96, 254], LA [254, 222], SA [144, 196, 222, 96], Apo-/lipoproteins: ApoE [95, 96, 94], ApoJ [94], ApoA-1 [94, 96], ApoA-II [94, 96], LDL, HDL [94, 144, 93], PAMP/DAMPs: [244, 245], (−) bacterial products (LPS, LTA) [244], Nucleic Acids [160], Heat Shock protien 60 [249], Apoptotic cells [95, 153, 160, 96, 222], Macropahages [252], Astrocytoma cells [244]

Despite substantial efforts, the identity of the biological ligands of TREM2 remains controversial (Fig. 4). TREM2 is known to modulate myeloid cell activity in response to microbial products [241], which led several groups to test bacteria as a possible source of TREM2 ligands. Using a TREM2-Fc fusion protein that consists of the extracellular ectodomain of TREM2 attached to the Fc-portion of human IgG, TREM2 was found to bind to some bacteria [242, 243], including Gram-positive (*S. aureus*) and Gram-negative species (*E. coli*, F. tularensis), but not *S. cerevisiae* [244, 245], Salmonella or Typinmurin [246]. TREM2 was specifically found to bind highly anionic bacterial products [244] and pertussis [247] and cholera [248] toxins. In addition to microbial products, TREM2 was also reported to bind to high molecular-weight nucleic acids [160] and heat-shock protein 60 [249], that were further shown to initiate TREM2 signaling in reporter cell lines. While some of these interactions were found to be of relatively low affinity [249], recognition of many molecules like carbohydrates and glycans induce only minimal biological signaling at low densities, but at high densities when receptors are forced into closer contact, can create strong biological effects [250]. Indeed, glycosaminoglycans and specifically heparin sulfate were found to modulate TREM2 binding [223] and it has been suggested that this may result in clustering of TREM2 on the membrane, thus potentially modulating TREM2 binding to other ligands [251].

Unidentified TREM2 ligands were also detected by TREM2-Fc binding to the cell surface of macrophages [252], human astrocytoma cells [244], dendritic cells [253], N2A cells [153, 223], THP-1 cells [223] and apoptotic cells [160]. In support of these findings, TREM2 deficiency partially impairs microglial recognition of apoptotic cells [94, 153, 160]. Recent studies have demonstrated that TREM2 binding to these cells is sensitive to proteinase K, suggesting the receptor binds to protein [223], perhaps complexed with proteoglycans.

It had previously been shown that members of the TREM family recognized lipid ligands [254], and lipids may also help mediate the interaction of TREM2 with ligands on the cell surface. Polar lipids found on the cell surface were assessed as possible TREM2 ligands [223, 242, 254], using lipid arrays [96] and reporter assays [196, 222]. While anionic lipids seemed to produce among the highest levels of response, additional factors that influence the particular lipids that TREM2 recognizes requires further study. This lipid binding may allow TREM2 to sense changes in the local environment as exemplified by TREM2-positive cells binding to externalized phosphatidylserine on apoptotic cells [153] and myelin debris [196].

Some studies suggest that TREM2 may bind lipids in cis with other protein-based interactions [223]. TREM2 has also been reported to bind to lipoproteins, including Apolipoprotein A1 (ApoA-I), clusterin (CLU), and low density lipoprotein (LDL), though ApoE has been most widely demonstrated to bind to soluble TREM2-Fc. This binding seems to occur independent of ApoE isoform [94–96, 255] and be dependent on residues 130–149 [255]. Because TREM2 binds to lipids, the lipidation status of ApoE and other apolipoproteins could dictate their binding affinity to TREM2. Several studies [95, 96, 255] demonstrate that TREM2-ApoE binding is not dependent on lipid loading. However, others have found that lipidation was necessary to drive TREM2 binding [94]. Lipid association is reported to be necessary for TREM2 binding to ApoE, ApoA-I and ApoA-II from cynomolgus macaque CSF and serum [96]. ApoE binding to TREM2 was found to induce TREM2 signaling in NFAT reporter cell lines [255], though how its binding to TREM2 would alter signaling in vivo remains to be determined. Because ApoE can bind to apoptotic cells [95] and amyloid plaques [95, 96], it has been proposed that an interaction between TREM2 and ApoE may indirectly allow it to mediate recognition and phagocytosis of these substrates. This may be important in the recognition of AD-related stimuli by TREM2, because TREM2 was found not to bind to plate-bound Aβ [222], but did bind to areas around amyloid plaques in an AD mouse model [158]. However, the possibility of TREM2 binding directly to Aβ, in addition to these indirect interactions, has not been fully excluded. Together, these data indicate that, in addition to protein – proteoglycan complexes, TREM2 may also bind to lipids and protein-lipid complexes.

AD-associated TREM2 variants including R47H, R62H, D87N and T96K, are found on the surface of the protein, and impact ligand binding [223]. Studies employing a TREM2 R47H-Fc chimeric protein revealed the R47H mutation significantly reduces TREM2 binding to cells [223], ApoE [96] including all three isoforms [95] and its lipidated form [94], other lipids [144, 222], apolipoproteins [94, 96] and lipoproteins [144]. TREM2 variants at either the same residue (R47A, R47E and R46A) [96] or with a similar R-to-H substitution (R62H) [94, 144] as R47H similarly disrupted TREM2 recognition of apolipoproteins [94] or cells [223]. However, R62H and R52H variants demonstrated relatively comparable lipid detection to WT TREM2 [144]. Other variants residing within the TREM2 ectodomain (Y38C, T66M, K48M) effectively abolished TREM2 binding to proposed ligands [94], while those located on the ectodomain proximal to the stalk region of TREM2 (D87N, T96K) exhibited enhanced interactions with some ligands [144, 223], while decreasing association with others [94]. TREM2 variants within the stalk region (H157Y, E151K, R136W) or intracellular domain (L211P) had no significant impact on ligand binding. The differences among these disease-conferring TREM2 variants’ recognition of cells, lipids, lipoproteins and apolipoproteins may alter how they impact downstream signaling. However, it remains unclear whether the ligands identified thus far are the relevant binding partners of TREM2 in vivo and mediate the receptor’s ability to respond to damage or infection in the CNS. Future work establishing the full array of physiological TREM2 ligands, and how variants impact these interactions will be instrumental in elucidating the role of TREM2 on myeloid cells in response to different pathologic stimuli.

### TREM2 associates with the intracellular adaptor DAP12

Because the major isoform of TREM2 has only a short cytoplasmic tail, it requires the intracellular adaptor DAP12 [136, 256] to mediate several of its signaling functions [139, 143]. Along with TREM2, DAP12 is required for signaling of other TREM family members [242], MDL-1, and Siglecs and can be used as an adaptor for other receptors critical for regulating myeloid cell function including CSF1R and toll like receptors [257]. In some contexts, cross-talk among these receptors has been shown to occur at the level of DAP12 availability.

In order to associate with the membrane, DAP12 requires the presence of its receptors [256]. Indeed, DAP12 is clustered at the same area of the membrane where TREM2 is highly upregulated on myeloid cell processes in contact with plaques in an AD mouse model [258]. At the membrane, DAP12 can associate with other DAP12 molecules to form homodimers through cysteine residues in its short extracellular domain [259]. In some contexts, DAP12 can also heterodimerize with DNAX activation protein of 10 kDa (DAP10) [260] which can modify downstream signaling cascades. It is not known whether DAP12 complexes constitutively associate with TREM2 or whether this is induced upon ligand binding in vivo but recent data using a split luciferase assay found that TREM2-DAP12 association in transfected HEK cells was primarily driven upon TREM2 stimulation [237]. Interestingly, the T66M TREM2 variant but not R47H or S116C variants enhanced the constitutive association with DAP12 in this system [237]. Regardless, activation of TREM2 and other DAP12-associated receptors results in tyrosine phosphorylation of DAP12 within its immunoreceptor tyrosine-based activation motifs (ITAM) by src family kinases casein kinase II at residues 85–88 and at residues 79–81 by PKC [259]. This phosphorylation occurs only when DAP12 is receptor-associated [139] and serine / threonine phosphorylation of the ITAM motifs are required for signaling [139].

### TREM2 signaling downstream of DAP12

These phosphotyrosine residues on the DAP12 ITAM serve as docking sites for a number of molecules that initiate signaling cascades that activate an immune response. There are several immune stimulating molecules that associate with DAP12 in response to TREM2 activation (Fig. 4). Crosslinking TREM2, commonly used to mimic TREM2 activation, can result in recruitment of DAP10, PI3K or LAB to the TREM2-DAP12 complex. In turn, these molecules are activated through tyrosine phosphorylation, principally by ITAM-associated Syk and go on to activate downstream signaling components, including Akt, Rac, Vav and MAPKs, including ERK [129, 136, 139, 159, 182, 260–263]. These initiate changes in gene expression and cytoskeletal rearrangement which mediate many downstream cellular functions associated with immune cell activation [264].

While ITAM domains are typically activating, they can be inhibitory in certain contexts [265, 266]. When the ITAM motifs of DAP12 are partially phosphorylated [267], inhibitory phosphatases SHIP, SHP and the adaptor downstream of kinase 3 (DOK3) are recruited to the TREM2-DAP12 signaling complex. These molecules inhibit immune activation [265, 268–270], possibly through blocking DAP10, PI3K, and Syk association with the TREM2-DAP12 complex and preventing activation of ERK, Vav3 and calcium mobilization [260].

Whether signaling through DAP12 results in activation or inhibition of the immune response seems to be receptor- and stimulus-dependent. Activation of TREM2 and myeloid DAP12-associating lectin 1 (MDL1) but not SIRPβ enhanced association of the inhibitory SHIP1 with DAP12 [260]. Similarly, macrophage colony stimulating factor (MCSF) alone but not MCSF and RANKL induced localization of SHIP1 to DAP12 [260]. Even different levels of the same stimulus can induce association of TREM2-DAP12 with different downstream signaling components. For example, Peng and colleagues [270] found that a low dose, but not a high dose of LPS resulted in association of DAP12 with DOK3. This served to dampen the cellular response to LPS, as DOK3 deficiency increased downstream signaling components, cytokine production and death of mice administered an otherwise sub-lethal dose of LPS. However, at high doses of LPS treatment, DOK3 did not associate with DAP12. SHIP1 was also shown to moderate the response of TREM2-induced proliferation. When SHIP1 deficient preosteoclasts were exposed to an activating TREM2 antibody, osteoclast formation was upregulated an additional 4-fold [260].

Whether DAP12 serves to activate or inhibit the immune response depends on the receptor it is associated with, the stimulus used to activate that receptor and the strength of that stimulus. It may also depend on availability of different downstream signaling components locally at the membrane, their relative expression in the cell [262] or other environmental factors [271]. Much of the signaling data thus far was performed in cultured osteoclasts, and future studies may find that other cell types use distinct signaling mechanisms. Whether TREM2 signaling is activating or inhibitory in the context of disease is also not known, and a greater understanding of the different TREM2 pathways that are relevant in disease will be instructive. Interestingly, SHIP1 variants also confer risk for AD, and it is thought that these variants result in a change in the transcriptional start site of SHIP1 resulting in a protein that lacks its SH2 domain which is necessary for association with ITAMs and immunoreceptor tyrosine-based inhibitory motifs (ITIMs) [272]. This suggests that these inhibitory components that associate with TREM2 deserve attention moving forward in understanding immune-related pathways that are important in AD.

### Other TREM2 signaling complexes

There is a general consensus that TREM2 likely acts as a homodimer or homomultimer to induce downstream signaling. This is a common mechanism of activation of other receptors with similar structures to TREM2 in which ligand binding induces complex formation and initiates downstream signaling cascades. Almost all studies examining TREM2 signaling have provoked TREM2 dimerization using antibody-mediated crosslinking to induce signaling [136]. However, there is also evidence that TREM2 can associate with other receptors, including PlexinA1 [273, 274]. Other studies are suggestive that TREM2 could also bind to TREML1 [236] through co-IP experiments or CSF1R due to their close linkage in network analyses [101] and the strong commonalities in their downstream pathways [222, 275]. While these last two interactions remain to be validated, it is certainly possible that TREM2 also acts through these alternative heteromeric complexes. Whether this is regulated by the cell type or context, and whether this is an important role of TREM2 endogenously in the context of NDDs, remain to be determined.

### Biological actions of sTREM2

The biological roles of sTREM2 have been controversial. Initially sTREM2 was postulated to act as a decoy receptor opposing full-length TREM2 function. A soluble version of a closely related TREM family member, sTREM1, modeled by a TREM1-Fc fusion protein, competes with its membrane-bound form to block TREM1 signaling [276] and produces opposing effects on inflammation and survival following LPS injection in mice [277]. Soluble TREM2 may similarly act as a decoy receptor to negatively modulate TREM2 signaling [198, 225]. In support of this, in vitro a chimeric TREM2-Fc protein used to model soluble TREM2 inhibited osteoclastogenesis, a process that requires TREM2-DAP12 signaling [278].

Recent studies suggest that sTREM2 may have its own biological function. Exogenously applied sTREM2 was internalized by cultured bone marrow derived macrophages, and promoted survival in cells lacking TREM2 expression [149]. However, sTREM2 failed to rescue phagocytosis in TREM2 deficient bone marrow macrophages in culture [279]. Recent data also demonstrate that treatment of microglial cell lines with TREM2-Fc or a HEK-cell produced sTREM2 peptide increases survival, in line with full-length TREM2 function [14]. This study also found that sTREM2 strongly induced an inflammatory response in culture models of microglia. These data suggest that there are important biological roles of sTREM2 other than acting as a decoy for the full-length TREM2 receptor. The mechanisms underlying sTREM2 function are not yet well understood, but interestingly, does not require the presence of full-length TREM2 or its intracellular adaptor DAP12 [14].

The signaling role of TREM2 CTFs is also starting to be explored. In the absence of γ-secretase activity, membrane-associated TREM2 CTFs have been proposed to either promote TREM2 anti-inflammatory signaling in response to LPS [280], or impair TREM2 signaling by sequestering DAP12 from interacting with full-length receptors [230], reducing DAP12 phosphorylation and downstream PLCγ activation [230, 232] along with TREM2-mediated phagocytosis [232]. Production or stabilization of CTFs on the membrane may also provide a point of cross-talk through which TREM2 could modulate the signaling of other DAP12-associated receptors. It is clear that further study will be necessary to gain insight into how these soluble TREM2 products impact signaling both of TREM2 and other pathways critical to innate immunity.

## TREM2 function

### TREM2 regulates myeloid cell number

While TREM2 expression and signaling are context-dependent, there are some commonalities in TREM2 function that have been found across the diverse cell types and environments in which it has been studied (Fig. 4), one of which is regulating myeloid cell number. The impact of TREM2 on myeloid cell number outside of the context of disease or stimulus is not completely clear. While knocking down TREM2 in primary microglia lead to reduced cell number [106], it had no effect on osteoclasts derived from peripheral blood mononuclear cells (PBMC’s) from PLOSL TREM2 E14X carriers lacking TREM2 expression [19] and microglial numbers were the same up to 1 year of age in mouse models lacking TREM2 expression. However, crosslinking TREM2 did promote an increase in osteoclast number in culture [143]. What is clear is that TREM2 has an effect on increasing myeloid cell number in response to inflammation or disease. TREM2 deficiency was shown to prevent increases in the brain myeloid cell populations in response to traumatic brain injury [98], ischemia [160, 193], aging [196], and in the initial response to demyelination [196], though it did increase the number of cells in a model of sepsis [170].

TREM2 deficiency also prevented local increases in myeloid cells around plaques in AD. Amyloid plaques are typically surrounded by a rapidly recruited [281, 282] cluster of activated myeloid cells in AD human brain tissue [283, 284] and in AD mouse models [285]. Recent evidence demonstrates reduced myeloid cell accumulation around amyloid plaques in TREM2 hemizygous [222, 258, 286], and TREM2- [145, 162, 222, 258, 287] and DAP12-deficient [258] AD mouse models, as well as in postmortem AD human brain tissue from individuals harboring the TREM2 R47H variant [60]. These data illustrate that TREM2, and its adaptor protein, DAP12, are required for myeloid cell accumulation around amyloid plaques. While Wang and colleagues [145, 222] found that TREM2 deficient AD mice had a decrease in total brain myeloid cells, others found that this was primarily driven by the specific loss of plaque-associated myeloid cells [258, 287]. Together, this suggests that TREM2 is important for myeloid cell expansion in response to disease. Evidence suggests that in various contexts, TREM2 is important for myeloid cell survival, proliferation and chemotaxis, all of which could lead to disease-associated increases in myeloid cell number.

### TREM2 enhances myeloid cell survival

TREM2 has been shown in multiple contexts to be important for cell survival. Osteoclasts [261] and bone marrow derived macrophages [149] from TREM2 deficient mice, and liver cancer [186] and glioma cell lines [183] in which TREM2 was knocked down had increased levels of caspase 3, Bcl-2-associated X protein (bax), Annexin V and TUNEL positivity, all suggesting that TREM2 deficiency enhanced apoptosis. Similarly, primary microglia and the BV2 microglial cell line with reduced TREM2 expression had decreased survival, along with decreased levels of elements of the survival-related Wnt/β-catenin pathway [13]. Conversely, TREM2 activation through receptor crosslinking increased survival of monocyte-derived dendritic cells [136] and osteoclasts [260]. In culture, microglia derived from TREM2 deficient mice did not show more cell death at baseline, but when levels of CSF1, an important factor for the maintenance of microglial survival, were reduced, TREM2 deficient microglia were more likely to undergo apoptosis [222]. This was also found to be the case in bone marrow derived macrophages [149]. In an AD mouse model, TREM2 deficiency also increased the number of plaque-associated myeloid cells which were TUNEL+ [222]. Taken together, these studies suggest that TREM2 is protective against apoptosis, especially under stressful cellular conditions.

### TREM2 enhances myeloid cell proliferation and differentiation

TREM2 may also increase cell number through promoting myeloid cell proliferation. In glioma cell lines [183], liver cancer cell lines [186], and primary microglia [13], reduced levels of TREM2 led to cell cycle arrest. The number of proliferating myeloid cells were also decreased in vivo in response to demyelination [288], colonic mucosal injury [189] and in AD mouse models [145, 287] lacking TREM2 expression. While the mechanisms of this regulation of proliferation are not clear, TREM2 deficiency in cultured osteoclast precursors prevented CSF1-mediated proliferation [261], a process also critical for proliferation of many macrophage populations, including brain myeloid cells. It has been suggested that TREM2 may interact with the CSF-1 receptor to mediate these effects. In dendritic cells derived from PLOSL patient PBMCs expressing Q33X and V126G TREM2 variants, gene expression profiling identified “negative regulation of proliferation” as a genetic pathway which was significantly increased in variant carriers compared to controls [135]. In addition to being important for directing proliferation of the cells in which it’s expressed, TREM2 might also promote a myeloid cell phenotype that directs proliferation of other cells in the surrounding microenvironment. TREM2 is highly upregulated during organogenesis when macrophages release factors to promote proliferation of surrounding cells [137], in tumor associated macrophages where analogous macrophage-driven trophic support occurs [183], and following CNS trauma where myeloid cells serve as an important source of neurotrophic support during tissue repair [289]. Interestingly, TREM2 is strongly upregulated by neural stem cells [290] and ESC-derived oligodendroglial precursors [291]. A relationship between TREM2 expression and neurogenesis has not yet been explored, but given the influence of TREM2 of proliferation on other cell types, this may warrant further examination.

TREM2 may also influence cell differentiation. Differentiation was impaired in osteoclasts derived from PLOSL patients expressing TREM2 variants [19] and in RAW macrophages deficient for TREM2 through a PlexinA1-dependent pathway [274]. However, Otero and colleagues [261] demonstrated that mouse-derived TREM2 deficient preosteoclasts differentiated into osteoclasts faster. Though the role of TREM2 in cell differentiation is not completely clear, this step in cell phenotype determination may also contribute to the changes in cell numbers and population observed in the context of TREM2 deficiency.

### TREM2 regulates myeloid cell chemotaxis

Another potential contributor to TREM2’s role in expanding the myeloid cell population in the context of disease or inflammation is by modulating chemotaxis or migration of these cells. In culture, knocking down TREM2 reduced chemotaxis of glioma cells in a Boydon chamber assay in response to serum [183], and in the BV2 microglial cell line in a scratch assay [158]. Microglia from TREM2 deficient brain slices exhibited reduced chemotaxis into co-cultured brain tissue from old or AD mouse models [292]. In addition, TREM2 deficient mice had fewer microglia migrate to the site of apoptotic neuron injection in the brain and had slower process extension toward a brain laser lesion as measured using two photon microscopy [292]. Conversely, TREM2 crosslinking increased CCR7-dependent chemotaxis [136, 139, 143]. TREM2 was also found to be co-enriched with genes involved in purinergic signaling, a key pathway directing microglial chemotaxis in network analyses [101], though whether TREM2 regulates P2R-receptor mediated chemotaxis has not been examined experimentally. However, others did not see a deficit in chemotaxis in PLOSL patient-derived osteoclasts [19]. Kiialainen and colleagues [135] found that PBMC’s cultured from patients with PLOSL-associated TREM2 variants had both up- and down-regulated components of the chemotactic response. It may be that different components of the chemotactic pathway and therefore different types of chemotaxis are differentially regulated by TREM2.

Studies have examined the effect of TREM2 on specific chemotactic pathways that are involved in tissue infiltration by myeloid cells. Wang and colleagues [183] found that TREM2 deficient glioma cell lines downregulated CXCL10, CXCR3, MMP2 and MMP9 which are all important in tissue invasion. In network analyses, TREM2 was significantly co-enriched with DOCK2 and DOCK8 which are involved in tissue transmigration [101] and mice deficient for TREM2 had reduced leukocyte infiltration following experimental induction of colitis [190]. There was also decreased neutrophil recruitment to mouse lungs in response to bacterial infection in TREM2 deficient mice [173]. In vitro*,* cells lacking TREM2 expression had reduced chemotaxis toward CCL2 [292]. Mice lacking DAP12 were found to have significantly reduced recruitment of peripheral macrophages in vivo in response to cigarette smoke or intranasal C-C motif chemokine ligand 2 (CCL2) administration, and these DAP12-chemotactic deficits were found to be rescued by reintroducing a TREM2-DAP12 fusion construct [141]. TREM2 deficient mice also had reduced levels of CCL2 [292] and fewer peripheral immune cells in their brain following middle cerebral artery occlusion (MCAO) [193]. There was a trend toward a correlation between CCL2 and sTREM2 levels in the CSF of human AD patients, which may suggest that TREM2 plays a role in mediating CCL2-mediated chemotaxis of cells in the context of AD as well. However, other studies have found no link between TREM2 and monocyte trafficking into inflammatory tissues [149]. Future studies will be necessary to assess which chemotactic pathways are influenced by TREM2 and whether that includes pathways related to peripheral immune cell infiltration into the CNS in NDDs.

### TREM2 regulates phagocytic function

A well-characterized function of TREM2 is to enhance phagocytosis. TREM2 is expressed in a subset of myeloid cells within the CNS that have high phagocytic capacity [215]. Across numerous in vitro studies, loss of TREM2 results in reduced phagocytosis of a variety of substrates, including apoptotic neurons or neuronal cell lines [95, 139, 153, 160], bone [19, 293], bacteria and bacterial products [170, 225, 237, 249] and lipids [94, 129]. Conversely, TREM2 activation or overexpression enhanced uptake of these substrates [139, 159, 214]. TREM2 expression correlated with Aβ40 uptake in BV2 cells in which TREM2 was knocked down or overexpressed [158]. Aβ42 uptake was also reduced in TREM2 deficient primary microglia [214, 225] and in the N9 microglial cell line expressing a non-functional TREM2 when plated onto brain slices from AD mouse models [279]. In agreement with these findings, in vivo*,* TREM2 deficient mice have reduced localization of Aβ within CD68+ phagosomes in AD mouse models [258] and reduced uptake of deposited Aβ three hours after injection into the brain [145]. Together, these findings suggest that TREM2 is important for Aβ uptake by brain myeloid cells. However, in culture, TREM2 expression was no longer found to correlate with Aβ uptake after pretreatment of cells with LPS [158]. A similar effect was observed in a mouse model of sepsis where injection of myeloid cells overexpressing TREM2 enhanced bacterial phagocytosis and survival, but not if the mice were pretreated with LPS [180]. These findings suggest that the mechanisms of TREM2-dependent phagocytosis can be modified by other signals in the microenvironment. Interestingly, the other modulatory components present in the brain microenvironment change throughout the course of NDDs, which could explain some of the differences in TREM2 function at different stages of disease progression. Outside of AD, TREM2 is important for clearance of myelin in experimental autoimmune encephalomyelitis (EAE) [159] and peri-infarct tissue in mice following MCAO [160]. However, it does appear that the effect of TREM2 on phagocytosis can be cell type specific. Sharif and colleagues [173] found that bone marrow macrophages derived from TREM2 deficient mice had reduced phagocytosis, but TREM2 deficient alveolar macrophages had increased uptake of bacteria in vitro and in vivo. R62H [94] and R47H TREM2 variants had impaired phagocytosis [240]. This was also true in HEK cells transfected with TREM2-DAP12 fusion constructs expressing R47H, T66M and Y38C variants [225]. Interestingly, while all of these variants impaired uptake of polystyrene beads, T66M and Y38C but not R47H impaired uptake of *E. coli* particles [225], suggesting that different TREM2 variants could affect recognition of specific phagocytic substrates as well as induce changes in basal phagocytic activity reflected in the fluid phase uptake of beads. More studies will be required to parse out the role of TREM2 in basal phagocytosis and cargo-driven phagocytosis of specific substrates.

The mechanism underlying TREM2-dependent uptake of various substrates is not clear. While transfection of a TREM2-DAP12 construct into CHO cells was shown to be sufficient for uptake of Neuro2A cells [153], it may be that TREM2 does not have to directly bind to its phagocytic substrates, as TREM2 binding to Hsp60 was sufficient to increase phagocytosis of bacteria [249]. If TREM2 does not directly bind to these substrates, then it must interact with other phagocytic pathways. It is possible that TREM2 impacts fluid-phase phagocytosis rather than cargo driven phagocytosis. TREM2 may also interact with other phagocytic receptors. For example, MerTK is essential for the phagocytosis of apoptotic cells [294] and is upregulated on the same cell population as TREM2 in AD [213]. Network analyses have also shown that TREM2 is co-enriched with genes involved in FCγR and complement-mediated phagocytosis [101]. In support of an association between TREM2 and Fc-dependent phagocytic pathways, stimulating cells lacking TREM2 function with an antibody against the desired phagocytic substrate did increase internalization of the substrate, but did not rescue it back to WT levels [279]. TREM2 deficient alveolar macrophages were found to increase phagocytosis and this was found to be dependent on the upregulation of first component of complement q (C1q) in these cells [173], which acts to opsonize phagocytic substrates. It may also be that sTREM2 plays a role in binding and directing phagocytosis of substrates by these other pathways, as ADAM inhibitors reduced sTREM2 production and decreased phagocytosis of *E. coli* [225].

Some have also suggested that these findings may reflect changes in degradation of phagocytic substrates rather than their uptake. Forabosco and colleagues [101] found that genes associated with lysosome activity were co-enriched with TREM2 across the brain and in monocyte-derived macrophages. In PLOSL patients, there is an accumulation of large CD68+ myeloid cells, suggesting that phagocytic uptake by these cells may be intact [25]. In a cuprizone model of demyelination, TREM2 deficiency was found not to impair uptake of myelin debris, but that this debris remained in cells longer than in controls, suggesting that degradation was specifically impaired [288]. This was also found to be true in TREM2 deficient macrophages which were able to take up bacteria at comparable levels to cells expressing WT TREM2 but were unable to kill and degrade them [263]. However, Jiang and colleagues [214] found that in primary microglia in which TREM2 was knocked down, Aβ42 degradation was unaffected. Together, the exact role of TREM2 in phagocytosis and other means of cellular uptake and degradation of substrates from the microenvironment remain unclear, though it clearly does play an important role in these processes.

### TREM2 modulates inflammatory responses

TREM2 interacts with many other inflammation-related pathways. While TREM2 has been touted as being anti-inflammatory, it seems that the interaction between TREM2 and other inflammation related pathways is actually more complex. Depending on the precise stimuli, the strength [260] and duration [4] over which they are presented, the cell type and the context, TREM2 can play different roles in the inflammatory response. In support of this, network analyses found that TREM2 was co-enriched with both classically pro- and anti-inflammatory gene clusters in the brain [101]. Likewise, a microarray analysis of macrophages derived from a PLOSL patient PBMC’s showed components of the inflammatory response and innate immune response were both up- and down-regulated, respectively, relative to controls [135]. Outside of the context of injury or disease, the transcriptional profiles of TREM2 deficient [222] or overexpressing [214] myeloid cells compared to controls was fairly similar. It is in the context of disease where TREM2 seems to heavily influence changes in inflammation-related pathways.

TREM2 has been classically described as being anti-inflammatory and several in vitro and in vivo studies are supportive of an anti-inflammatory role for TREM2 in certain contexts. Knocking down TREM2 in cell lines increases levels of proinflammatory mediators such as iNOS, TNFα, IL1β and IL6 [240] in response to apoptotic neuronal membrane components [139], TLR ligands [168], including LPS [159, 169, 170, 280] and Aβ42 [157]. A transient knock down of TREM2 in the P301S tau model and in the SAMP8 model of accelerated aging also increased inflammatory cytokine production [201, 216]. TREM2 deficiency also resulted in increased levels of IFNγ, TNFα and iNOS [189] following colonic mucosal injury and TREM2 knockdown or antibody-mediated inhibition increased expression of many inflammation-related cytokines following corneal infection [182]. Moreover, overexpressing TREM2 in cell lines, amyloid [214] and tau models of AD [295] reduced levels of these pro-inflammatory transcripts. Together, these studies suggest that in some contexts, TREM2 can attenuate inflammatory responses.

However, many other studies also support that TREM2 can mediate or amplify inflammatory responses. For instance, TREM2 knockdown impaired ROS production [246, 263]. TREM2 deficient microglia are more ramified in culture, a morphological signature of reduced activation [160]. TREM2 deficient AD mouse models have reduced levels of inflammation-related transcripts in both unbiased RNA sequencing approaches [222] and in the genes IL1β and IL6 in targeted analyses [162, 287]. Plaque-associated cells in AD mouse models deficient [222] or haploinsufficient [286] for TREM2 also had decreased cell soma size, surface area and increased process length, indicative of reduced activation [222]. Recent work using single cell sequencing approaches indicates that TREM2 is required specifically for a second phase of the myeloid cell response in AD which allows cells to fully adopt a neurodegeneration-associated phenotype [296]. This may be true in diverse disease contexts as pro-inflammatory cytokine levels were also reduced in TREM2 deficient mice following traumatic brain injury [98], ischemia [193], lung infection [149, 173] and demyelination [196], where TREM2 deficient brain myeloid cells exhibited a less activated morphology [288]. Conversely, activation of TREM2 in a macrophage cell line increased NO release [136], agonizing TREM2 following spinal nerve transection increased TNFα and IL1β [194] and overexpression of TREM2 increased expression of IL6, TNFα and MCP1 in mouse adipose tissue [129]. Because these studies examined gene expression in whole tissue, it is not clear whether these changes are due to changes in immune cell phenotype or alteration in cell number in the affected tissues. However, taken together, these findings clearly indicate that TREM2 can also promote inflammatory responses in certain contexts. This body of data strongly opposes the often-cited descriptor of TREM2 as an anti-inflammatory receptor. Future studies will be required to delineate the molecular and environmental determinants that govern how TREM2 contributes to the inflammatory response in different contexts.

While the number of studies have been limited, TREM2 variants associated with NDDs also seem to have mixed effects on inflammatory responses. The R47H variant impaired inflammatory responses in BV2 cells [240], yet R47H carriers with AD had increased expression of genes related to inflammatory pathways compared to non-carriers [57]. A variant within intron 2 of TREM2, which is prevalent in African American individuals, was found to be significantly associated with levels of C-reactive protein (CRP), a systemic marker of inflammation, whose expression is primarily driven by IL6 and IL1β [110]. However, it is not clear whether this represents a direct relationship between this TREM2 variant and systemic inflammation.

In addition to impacting the inflammatory responses of myeloid cells, TREM2 also seems to be able to indirectly feedback onto the inflammatory response in other cells within the microenvironment, including astrocytes. Astrocytosis, measured by glial fibrillary acid protein (GFAP) levels, was reduced across all stages of pathology examined in TREM2 deficient AD mouse models [162, 287], in areas of active demyelination [288] and trended toward a reduction in GFAP area in mice following ischemia [160]. However, GFAP levels were unchanged in TREM2 deficient mice at acute and chronic time points following traumatic brain injury [98], suggesting that TREM2 must work in tandem with context-dependent signals to alter astrocyte activation. One of the characteristic features of PLOSL is astrocytosis [22] and in a PLOSL patient with a TREM2 variant, GFAP levels were significantly increased in frontal lobe tissue [28]. This suggests that TREM2 can play multiple roles in regulating astrocyte activation depending on the precise context.

### Other functions of TREM2

While regulating cell number, phagocytosis and inflammation are the best studied roles for TREM2, other studies have suggested additional roles for the receptor, such as regulation of synaptic pruning and monitoring of synaptic function [297]. Because of the cross-talk between TREM2 and complement pathways and a clear role of TREM2 and complement in phagocytosis in disease [298], it would be of interest to assess whether TREM2 influences synaptic function by modulating synaptic pruning, either normally during development or aberrantly in the context of NDDs. Others have suggested that, due to the close apposition of TREM2+ cells to oligodendrocyte precursors during development, they may support their function [154]. TREM2 has also been shown to be important for angiogenesis following stroke [160]. Because of TREM2’s proposed lipid-related ligands, and the strong links between lipid metabolism and NDDs, it would not be surprising if TREM2 also played roles in this pathway. In support of this, lipid metabolism was the most strongly altered pathway in TREM2 deficient mouse brains following cuprizone-induced demyelination [288]. How TREM2 affects these normal functions within the brain has not been studied, but may represent important future areas of investigation.

Outside of the brain, studies have proposed additional roles for the TREM2 receptor. TREM2 has been proposed to play a role in adaptive immunity. Myeloid cells expressing higher levels of TREM2 were able to increase T cell proliferation better than those expressing lower TREM2 levels [158]. TREM2 was also found to be co-enriched with genes related to adaptive immunity in gene network analyses [101]. However, others have found that activating TREM2 through crosslinking did not upregulate molecules involved in antigen presentation [159, 299], suggesting that TREM2-mediated stimulation of adaptive immune responses may be indirect or require additional environmental factors. TREM2 also seems to be important for cell maturation [136] and in particular multinucleation of osteoclasts [19, 143, 293]. It is not yet known how TREM2 might mediate these additional functions.

## TREM2 and NDD pathology

### TREM2 impacts amyloid pathology in AD

The observed changes in TREM2 expression, signaling and function with disease-associated genetic variants ultimately translate to changes in NDD pathology. Many studies have focused on how TREM2 and disease-associated variants impact AD-related pathologies. Studies examining loss of TREM2 function in amyloid mouse models initially appeared to support contradictory conclusions. Some groups found that TREM2 deficiency reduced [162, 258] while others found that it increased [222] amyloid pathology. However, recent evidence has harmonized these results by demonstrating that TREM2 deficiency has a changing role throughout AD progression, reducing amyloid pathology early but increasing it at later stages of disease [287]. Studies overexpressing TREM2 in AD mouse models also found a temporal effect of this overexpression, which reduced pathology early in disease progression [214] but no effect at a later time point [300]. This is supported by Korvatska and colleagues [60] who demonstrate accelerated disease progression in R47H carriers compared to non-carriers with AD. This may also explain discrepancies in human studies of R47H carriers who found no association between R47H carriers and non-carriers in amyloid deposition [49] and others who found that R47H carriers had significantly more plaques compared with non-carriers [57]. This changing role for TREM2 throughout progression of amyloid pathology may also reflect a dynamic role for myeloid cells themselves. As Hickman and El Khoury [11] posit, these brain myeloid cells may be protective early through clearance of Aβ, but detrimental later in disease progression when they enhance the inflammatory response without being effective phagocytes. Alternatively, it could reflect how TREM2 impacts the phenotype and abundance of distinct myeloid cell subsets, or perhaps other microenvironmental cues which change TREM2 downstream signaling to favor alternative pathways. While mouse models so far have recapitulated several aspects of TREM2 localization and function observed in human brain tissue, it is worth noting that there is a caveat to studying TREM2 in mouse models of AD with PSEN mutations since γ-secretase is also important for TREM2 CTF cleavage [232]. It is not clear whether changes in γ-secretase activity would be likely to greatly alter TREM2 function in vivo*,* but this should be a consideration when interpreting these studies.

Interestingly, there also appears to be a difference in the mechanism by which TREM2 affects pathology early and late in disease progression. Early, TREM2 deficiency decreases the number of plaques, while later in disease progression, increase in pathology is instead driven by increased plaque size [287]. While it’s not clear exactly how TREM2 could modulate Aβ proteostasis early in disease progression to impact plaque number. TREM2 was shown to impact APP processing in a genome-wide siRNA screen [301], though since it is not neuronally expressed, this would likely occur through indirectly altering neuronal phenotype. Conversely, later in disease, the association of myeloid cells with plaques has been proposed to limit plaque growth by forming an insulated microenvironment or barrier [258, 302]. TREM2 deficiency appears to impede the formation of this barrier, and in doing so, cause a shift from compact to diffuse plaques [258].

### TREM2 modulates neuritic dystrophy in AD

TREM2 has also been studied in AD in the context of modifying neuritic dystrophy. Several studies have found increased neuritic dystrophy around plaques in TREM2 deficient mice [145, 258] and in human R47H carriers with AD [57]. TREM2 overexpression in 7-month but not 18-month-old APP/PS1 mice had increased levels of synaptophysin, suggesting that enhanced TREM2 expression may protect against Aβ-driven synapse loss [157, 300]. One possible mechanism for this lies in the larger, more diffuse plaques with high soluble Aβ affinity [258] observed in TREM2 deficient mice late in disease progression [287]. The relative toxicity of soluble Aβ is well documented, including its roles in blocking long-term potentiation [303] and inducing tau hyperphosphorylation and aggregation [304, 305]. Together, these data suggest that functional TREM2 is necessary for microglial clustering around amyloid plaques and may thereby form a barrier around plaques which limits neuritic dystrophy. However, not all data support a protective role of plaque-associated myeloid cells on AD pathology. Microglia can serve as synaptotoxic agents in AD through complement-mediated synaptic pruning [298]. In this way, the loss of plaque-associated myeloid cells due to TREM2 deficiency could be beneficial. Others suggest that it may not be that more dystrophic neurites are formed around plaques in TREM2 deficient mice and R47H carriers, but that TREM2 deficient myeloid cells are not as effective at clearing them [215]. Further evidence will be required to assess the formation and clearance of dystrophic neurites across stages of AD in the context of TREM2 deficiency or TREM2 variants to assess these possible mechanisms.

### TREM2 affects tau hyperphosphorylation and aggregation in AD

The impact of TREM2 on tau pathology in AD has also been examined. The effect of TREM2 on phosphorylated tau (p-tau) accumulation in dystrophic neurites in AD is not clear, with some studies showing an increase [145, 222] and others showing a decrease [162] in hyperphosphorylated tau markers surrounding plaques in TREM2 deficient amyloid mouse models of AD. These different outcomes are likely related to disease progression dependent effects on the amyloid pathology driving this accumulation. Less work has been done in tau models of AD, but overexpressing TREM2 under the CD11b promoter in the P301S tau model of AD resulted in reduced hyperphosphorylated tau levels, coordinate with a decrease in activation of two of the known tau kinases, cyclin dependent kinase 5 (CDK5) and GSK3β [295]. Opposite effects were observed in P301S mice in which TREM2 was knocked down [216]. In humans, TREM2 protein levels in the temporal cortex of AD patients correlated with tangle score and paired helical filament (PHF) levels [164] and sTREM2 levels in CSF are correlated with CSF tau levels early in clinical AD progression [202], suggesting an important relationship between TREM2 and tau pathology in humans. R47H patients had higher levels of CSF p-tau [53, 61], and a variant located upstream of TREM2 was associated with increased tau pathology in the brain [109]. Together, these findings suggest that TREM2 variants may also have an impact on tau-related pathologies in AD, though the mechanisms governing this association are less clear.

### TREM2 affects synaptic and neuronal loss in AD

Studies have also examined how TREM2 and its variants impact neuronal and tissue loss and cognition in AD. TREM2 protein levels in the temporal cortex of AD patients were positively correlated with cleaved caspase 3 and negatively correlated with the presynaptic marker SNAP25 [164], suggestive that loss of TREM2 could impact synapse pathology. There was also a significant reduction in neurons in layer V of the cortex in TREM2 deficient amyloid models of AD [222] and a substantial rescue of neuronal loss when TREM2 was overexpressed in amyloid [157] and tau [295] AD mouse models. These changes in neuron number also correlated with a rescue in behavioral deficits [157, 295]. Interestingly, however, TREM2 expression levels on peripheral monocytes correlated with lower MMSE [128] and MoCA scores as well as reduced gray matter volume [148]. R47H variant carriers with AD also had reduced gray matter volume [63] in the temporal cortex and hippocampus [61]. There was also a trend toward a reduction in hippocampal volume [306] and significant decreases in other brain regions [63] in R47H carriers even in the absence of clinical AD. Though no changes in cognitive function were reported in middle-aged R47H carriers [307], the variant did correlate with cognitive deficits in older adults with R47H variants [6]. This was also true in healthy individuals heterozygous for NHD variants [308]. These findings suggest that TREM2 variants may have direct effects on neuronal loss, even in the absence of AD pathology.

### The effect of TREM2 on other NDD pathologies

Although not as extensively studied as in AD, the impact of TREM2 on other NDD-related pathologies has also been assessed. PLOSL patients have severe white matter dystrophy [22] and oligodendrocytes that survive in PLOSL patient white matter express markers of cell stress [309] suggesting a role for TREM2 deficiency in white matter degeneration. In cuprizone-mediated demyelination models, TREM2 deficiency impaired recovery and increased levels of axonal degeneration markers [288, 289]. In addition, injecting mice with TREM2 transduced myeloid cell precursors prevented EAE-induced demyelination and ameliorated motor phenotypes [159]. In addition to affecting oligodendrocyte survival and recovery following demyelination, TREM2 variants in PLOSL have also elucidated other roles of TREM2 in NDDs. PLOSL patients often experience seizures [22, 310], resulting in excitotoxicity. One patient with a predicted loss-of-function TREM2 PLOSL mutation had a reduction in many synaptic components, including nine GABA receptor subunits, which could play a role in mediating this enhanced excitability [28]. However, the mechanism underlying this phenomenon is not well understood and requires further study.

### The effect of TREM2 on inflammation-related pathologies

TREM2 has also been shown to modify tissue loss and behavior in several neuroinflammatory contexts. TREM2 deficiency reduced hippocampal volume loss and improved some behavioral outcomes at chronic time points following traumatic brain injury [98]. In contrast, TREM2 deficient mice had increased infarct volume in one MCAO model [160], though no change in another study [193]. Treatment with a TREM2 agonist induced pain behavior in mice in a DAP12-dependent manner even in the absence of nerve injury [194]. Outside of the brain, TREM2 deficiency results in increased body weight and glucose and insulin intolerance in mice fed a high fat diet [129]. TREM2 variant carriers may also have increased risk of systemic infection [311] and TREM2 deficiency is detrimental in the context of bacterial infection [149, 170, 180, 263]. Overall, in the brain and the periphery, despite great advances in assessing how TREM2 alters pathology, there is still no clear picture of how TREM2 mediates these diverse functional impacts across inflammatory and disease contexts. This will require a greater understanding of TREM2 expression, signaling and function and how these features change in the context of pathology. While these studies do point toward some common mechanisms by which TREM2 might modify aspects of pathology relevant to multiple NDDs, it is clear that the role of TREM2 in NDDs is not simple.

## The clinical relevance of TREM2

### TREM2-related biomarkers

Since the identification of NDD-associated TREM2 variants, and the detection of sTREM2 in the CSF and plasma of AD patients, there has been much excitement about how this may translate into immune-related NDD biomarkers and therapeutics. Elevated levels of sTREM2 were first detected in the CSF of patients with multiple sclerosis (MS) and other inflammatory neurologic diseases [312] and were found to be significantly elevated in MS patients [313]. These findings served as an impetus to examine whether CSF sTREM2 might also be changed in AD patients. Groups have reported elevations [314], reductions [225] or non-significant changes [203] in CSF sTREM2 in AD cohorts not stratified by disease stage (Table 1). However, as shown in Table 1, studies that did divide subjects by stage of disease progression, CSF sTREM2 levels were found to be significantly higher in patients with AD-related mild cognitive impairment [202, 315, 316] and mild dementia [202, 225] compared to controls and AD cases [202]. Cross-sectional studies in patients with dominantly inherited AD confirmed a significant increase in CSF sTREM2 starting 5 years before expected onset of clinical dementia, but found no significant differences between AD patients and controls beyond 5 years after symptom onset [12]. Together, these studies suggest a specific elevation in CSF sTREM2 levels in the early symptomatic stages of AD. Interestingly, sTREM2 was also found to be significantly elevated specifically in early stages of ALS pathology before returning to baseline at late stages of disease [317]. While it’s not clear whether these disease-stage dependent effects are due to a common mechanism, it will be interesting to see whether this pattern continues to be consistent across NDDs.

**Table 1:** Characteristics of experimental design for studies that examined sTREM2 in CSF and plasma

Study	Sample Type	Method of sTREM2 Detection	Main Finding (*p-*value)	sTREM2 (pg/ml), mean ± sd or median(IQR)	Study Cohort	Controls (CTR)	Diagnostic Parameters			Age (yrs) mean ± sd or Median(range)	Gender (percent female)	ApoE ε4+ carriers
							Criteria	Cognitive Testing	Other			
Gispert et al. (2016) [316]	CSF	ELISA	↑ sTREM2 [CTR-MCI (0.01); PreAD-MCI (0.036); CTR-AD (0.044)]	*Relat. to IS* CTR: 400 ± 200 PreAD: 530 ± 390 MCI: 710 ± 420 AD: 620 ± 400	PreAD (*n =* 19), MCI due to AD (*n =* 27), and mild AD (*n =* 23); all non-*TREM2* variant carriers	Cognitively normal subjects defined by MMSE score > 27, CDR = 0, and negative AD CSF profile^a^ (*n =* 45)	NIA-AA for AD and MCI due to AD groups	MMSE and CDR; PreAD had MMSE score > 27 and CDR = 0	MRI; Positive AD CSF profile: Aβ42 (<500 pg/ml), t-tau (>450 pg/ml), and p-tau (>75 pg/ml)	CTR: 60.98 ± 6.83 PreAD: 68.53 ± 7.93 MCI: 70.30 ± 7.35 AD: 66.78 ± 9.75	CTR: 63% PreAD:68% MCI: 55% AD: 69%	CTR: 15% PreAD:42% MCI: 52% AD: 48%
Suárez-Calvet et al. (2016) [202]	CSF	ELISA	↑ sTREM2 [CTR-MCI (0.002); AD-MCI (0.013); PreAD-MCI (0.062)]	*Relat. to IS* CTR: 470 ± 202 PreAD: 644 ± 410 MCI: 802 ± 380 AD: 725 ± 440	PreAD (*n =* 63), MCI due to AD (*n =* 111), and AD (*n =* 200), from five centers	Asymptomatic cognitively normal subjects with a negative AD CSF profile^a^, determined across five centers (*n =* 150)	NIA-AA for each group	NR	Positive AD CSF profile^a^ with cut-off values unique to each center (refer to [322])	CTR: 62.4 ± 11 PreAD: 70.8 ± 11 MCI: 74.3 ± 9 AD: 73.8 ± 10	CTR: 59% PreAD:60% MCI: 60% AD: 62%	CTR: 21% PreAD:58% MCI: 52% AD: 62%
Suárez-Calvet et al. (2016) [12]	CSF	ELISA	↑ sTREM2 [NC-MC (0.004)]	NC: 2824 ± 1292 MC: 3561 ± 1661	Cases with ADAD (*n =* 127) from DIAN cohort	Noncarriers (*n =* 91) for ADAD-related mutations from DIAN cohort	Presence of ADAD mutation (*PSEN1, PSEN2,* or *APP*) assessed by DIAN	MMSE and CDR	AD CSF profile examined	NC: 39.5 ± 11 MC: 40.4 ± 11	NC: 54% MC: 50%	NC: 34% MC: 28%
			↑ sTREM2 [MC with CDR = 0.5 (0.006); MC with CDR = 1 (0.044)]		No. of Subjects per CDR: CDR *n* 0 52 0.5 51 1 16 2 to 3 8	No. of Subjects per CDR: CDR n 0 88 0.5 3 1 0 2 to 3 0						
Heslegrave et al. (2016) [314]	CSF	Novel reaction monitoring assay with UPLC/TQ-S MS	↑ sTREM2 [CTR-AD (0.0457)]	CTR: 195.6 (131.0–240.7) AD: 231.2 (172.5–305.4)	*UK cohort:* AD (*n =* 37)	Cognitively normal with negative AD CSF profile^a^ (*n =* 22)	Revised IWG2 for AD	MMSE	Positive AD CSF profile^a^ (Aβ42 < 550 pg/ml, t-tau >375 pg/ml, p-tau >52 pg/ml)	CTR: 69.2 ± 8.0 AD: 70.51 ± 7.5	CTR: 45% AD: 53%	CTR: 33% AD: 67%
			↑ sTREM2 [CTR-AD (0.0312)]	CTR: 171 (153.5–241.5) AD: 230 (166.5–297.4)	*Swedish cohort:* AD (*n =* 24)	Cognitively normal subjects (*n =* 16)	NIA-AA for AD or MCI due to AD	MMSE	MRI or CT; Blood screening (unspecified)	CTR: 55.6 ± 9.7 AD: 64.3 ± 6.8	CTR: 56% AD: 54%	CTR: 31% AD: 69%
Kleinberger et al. (2014) [225]	CSF	ELISA	↓ sTREM2 [CTR-AD (0.001)]	*Relat. Values* CTR: 0.381 (0.283–0.518) AD: 0.309 (0.178–0.436)	AD (*n =* 56), from AD-confirmed cases across six centers	Cognitively normal with negative AD CSF profile^a^ (CSF: *n =* 88; plasma: *n =* 86)	NINCDS-ADRDA for probable AD	NR	Positive AD CSF profile^a^ defined by the Mattsson et al. equation: [324] (Aβ42/p-tau)/(3.694 + 0.0105 x t-tau)	CTR: 60.7 ± 9.5 AD: 70.4 ± 8.9	CTR: 63% AD: 68%	NR
	Plasma		↔ sTREM2 [CTR-AD (0.872)]	*Relat. Values* CTR: 1.022 (0.675–1.864) AD: 0.998 (0.702–1.375)	AD (*n =* 51), from AD confirmed cases across six centers					CTR: 60.4 ± 9.5 AD: 70.7 ± 9.0	CTR: 64% AD: 71%	NR
Henjum et al. (2016) [203]	CSF	ELISA	↔ sTREM2 [CTR-MCI (0.42); CTR-AD (0.17)]	CTR: 4400 (3000–5700) MCI: 4100 (2400–5900) AD: 4800 (3500–7100)	*Norwegian cohort:* AD (*n =* 29) or MCI due to AD (*n =* 21)	Cognitively normal subjects (*n =* 50)	DSM-IIIR for dementia; NINCDS-ADRDA for AD	MMSE	MRI or CT	CTR: 66 (50–86) MCI: 67 (55–75) AD: 68 (56–75)	CTR: 50% MCI: 57% AD: 45%	CTR: 0% MCI: 38% AD: 34.5%
			↔ sTREM2 [CTR-AD (0.76)]	CTR: 3200 (2800–5000) AD: 3800 (2600–5600)	*Swedish cohort:* AD (*n =* 25)	Cognitively normal with normal Aβ42 levels (>550 pg/ml) (*n =* 25)	NIA-AA for AD or MCI due to AD	MMSE	MRI or CT; Blood screening (unspecified)	CTR: 62 (43–80) AD: 79 (61–86)	CTR: 68% AD: 72%	CTR: 4% AD: 16%
Piccio et al. (2016) [86]	CSF	ELISA	↑ sTREM2 [CTR-AD (0.015)]	CTR: 1028 (244–2570) AD: 832 (163–2196)	Those with mainly mild AD, non-*TREM2* variant carriers (CSF: *n =* 73; plasma: NR), from two centers	Cognitively normal subjects (CDR 0) and negative for AD CSF profile^a^ (CSF: *n =* 107; plasma: NR)	NINCDS-ADRDA for probable AD	CDR	NR	CTR: 70.2 ± 8.5 AD: 76.6 ± 5.2	CTR: 53% AD: 49%	CTR: 48% AD: 64%
	Plasma		↔ sTREM2 [CTR-AD (0.74)]	CTR: 976 (65–2477) AD: 1019 (190–2546)						NR	NR	NR

*Abbreviations*: *AD* Alzheimer’s disease, mild cognitive impairment [313], *CSF* cerebrospinal fluid, *Aβ42* amyloid-β 1–42, *p-tau* Phosphorylated tau, *t-tau* total tau, *IS* internal standard, enzyme-linked immunosorbent assay [228], *UPLC/TQ-S MS* Tandem Quadrupole Mass Spectrometry, *NC* noncarriers, *MC* mutation carriers, *ADAD* autosomal dominant AD, *NR* not reported, *PSEN* presenilin, *APP* amyloid precursor protein, *PreAD* Preclinical AD, *DSM* Diagnostic and Statistical Manual of Mental Disorders, *NIA-AA* National Institute on Aging--Alzheimer’s Association, *NINCDS-ADRDA* National Institute of Neurological and Communicative Disorders and Stroke and the Alzheimer’s Disease and Related Disorders Association, *IWG2* International Working Group, *CDR* clinical dementia rating, *MMSE* mini-mental state examination, *MRI* magnetic resonance imaging, computed tomography, Dominantly Inherited Alzheimer Network [268]

^a^The ‘AD CSF profile’ consists of high t-tau and p-tau, and low Aβ42 levels in CSF; cut-off values are specified for each study. Under main finding, the results are presented as significantly elevated (↑), significantly reduced (↓), or non-significant differences (↔) in sTREM2

The detection and reported changes with disease progression in CSF levels of sTREM2 have raised questions of its origin and biological meaning in health and disease. In AD patients, CSF sTREM2 was not associated with changes in CSF Aβ42 [86, 202, 314, 316] but positively correlated with CSF biomarkers total tau [86, 202] and phosphorylated tau [202, 314, 316], including in cross-sectional cohorts with dominantly inherited AD [202]. Several groups have proposed that CSF sTREM2 levels may signify microglial activation in response to AD-related pathology [86, 202, 313, 314, 316]. Evidence of CSF sTREM2 positively correlating with glial protein YKL-40 in CSF [314, 316], another proposed AD immune biomarker, in addition to immunosuppressive agents causing a reduction in CSF sTREM2 levels [313] are consistent with this theory. Changes in sTREM2 appear to be independent of ApoE4 status [318–320]. Further work assessing how sTREM2 generation changes with microglial phenotype will be needed to definitively validate sTREM2 as an indicator of microglial activation. A recent clinical study proposes a neuroprotective role for sTREM2, reporting higher gray matter volume in areas susceptible to AD pathology for mild cognitive impairment (MCI) and AD patients with high CSF sTREM2 levels [316] though this correlation was not found in all studies. Higher levels of CSF sTREM2 at late stages of ALS also correlated with longer survival [317]. These findings, in combination with the positive correlation between CSF tau and sTREM2, could indicate that elevated sTREM2 production occurs as a protective response to neurodegeneration, though there is no definitive consensus yet as to the biological significance of sTREM2.

The current data on sTREM2 illustrate limitations for its use as an NDD biomarker. Several NDDs including MS, AD and FTD, are associated with elevated sTREM2 in CSF [86, 314], suggestive of a common innate immune mechanism in these distinct pathologies. It has also been suggested as a biomarker for welding fume exposure [142] and, as discussed above, can be regulated in many other inflammation-related contexts. This lack of disease and context specificity in sTREM2 changes raises concerns for its utility as a diagnostic tool for AD. Recent data show CSF sTREM2 levels are altered differentially throughout AD progression. These observations raise questions about its utility as a diagnostic readout for AD disease status in diverse neurologic cohorts, though does suggest that sTREM2 levels could be helpful in identifying stage of AD in coordination with other biomarkers [321]. Moreover, Heslegrave and colleagues [314] acknowledged that sTREM2 levels in AD patients and controls, while significantly different, substantially overlap, thereby further limiting its diagnostic utility. Transcriptome-based studies found dysregulation of several innate immune genes in blood from AD patients [320], which led others to assess whether TREM2 expression might also be changed in blood. While studies did find a correlation between TREM2 expression on blood monocytes and an AD diagnosis [128], sTREM2 in plasma was shown to not correlate with CSF sTREM2 levels [86] and plasma samples yielded non-significant differences in sTREM2 of AD and FTD patients compared to healthy controls [86, 225]. While sTREM2 alone does have clear limitations as a biomarker, it may have a potential application as part of a biomarker panel to assess the immune response in AD and other neurodegenerative diseases.

### TREM2-directed therapeutics

In addition to its potential as a biomarker, many have suggested that TREM2-directed therapeutics may prove to be a novel target for NDDs. There are several factors to consider in developing TREM2 therapeutics. First, while TREM2 variants confer as strong a risk for developing AD as one copy of the ApoE4 allele, the minor allele frequency of TREM2 variants are substantially lower, with less than 1% for TREM2 to approximately 20% for ApoE4 [7, 55]. Thus, though some have suggested that therapeutics might want to restore WT TREM2 function in these variant carriers as a potential therapeutic, correcting TREM2 variants are not likely to be a broadly applicable therapeutic approach. Rather, studying TREM2 variants that confer risk for NDDs will illuminate components of the immune response centrally important in immune modulation of pathology, and serve as a prerequisite to developing targeted immune-directed therapeutics. So far, the field has identified potential changing roles for immune cell function throughout progression of AD, and possibly identified a key role for peripherally derived immune cells in AD pathology, which would greatly aid in therapeutically targeting the immune cells relevant to AD pathology.

Common functions of TREM2 have been identified across multiple NDDs which suggest therapeutic targets could be relevant to multiple disease contexts. However, we have not found a simple explanation for what TREM2 does across cell types and contexts. Based on its disease progression dependent effects, it does not appear that simply activating or inhibiting TREM2 would be beneficial even in the context of AD. There may also be sex-dependent effects of TREM2, as some [86] but not all [202] have shown differences in sTREM2 levels in CSF between male and female subjects. Likewise, a TREM2 variant was associated with markers of systemic inflammation specifically in women not men, and was hormone-independent [110]. With the lack of strong biomarkers to stage NDDs and the variability in clinical progression among patients, it is not likely that increasing or decreasing TREM2 will be the universal solution to NDD pathologies.

## Conclusion

Rather, understanding when, where and how TREM2 is working is more likely to provide insights into immune function that can be modulated throughout disease progression. However, the emphasis on understanding TREM2 in NDDs began just 4 years ago and we still have a long way to go to understand TREM2’s expression, signaling, function and effects on these various pathologies. It will also be essential to start to dissect how the diverse array of TREM2 variants result in NDD risk. While this understanding of TREM2 variants may not directly translate into TREM2-directed biomarkers or therapeutics at this time, the insight into how the immune system actively participates in NDD pathology promises to provide many avenues for a new class of immune-directed therapeutic targets for NDDs.

## Abbreviations

5hmc: 5-hydroxymethylcytosine
AD: Alzheimer’s disease
ADAM10: A disintegrin and metalloproteinase domain-containing protein 10
Akt: Protein kinase B
ALS: Amyotrophic lateral sclerosis
ApoA: Apolipoprotein A (1, 2)
APOE: Apolipoprotein E
APP: Amyloid precursor protein
Aβ: Amyloid-beta (40, 42)
BAX: Bcl-2-associated X protein
C1q: First component of complement (C1), q
CCL2: C-C motif chemokine ligand 2
CCR: C-C chemokine receptor type (2, 7)
CD33: Siglec-3
CD68: Cluster of differentiation 68
CDK5: Cyclin dependent kinase 5
CHO: Chinese hamster ovary
CJD: Creutzfeldt-Jakob disease
CLU: Clusterin, or Apolipoprotein J
CNS: Central nervous system
CpG: [5′-C-phosphate-G-3′]
CRP: C-reactive protein
CSF: Cerebrospinal fluid
CSF1: Colony stimulating factor 1
CSF1R: Colony stimulating factor 1 receptor
CTF: C-terminal fragment
CXCR3: C-X-C motif chemokine receptor 3
CXCL10: C-X-C motif chemokine ligand 10
DAP10: DNAX activation protein of 10 kDa
DAP12: DNAX activation protein of 12 kDa
DOCK: Dedicator of cytokinesis (2, 8)
DOK3: Downstream of kinase 3
EAE: Experimental autoimmune encephalomyelitis
ER: Endoplasmic reticulum
ERK: Extracellular signal-regulated kinase
Fc: Fragment crystallizable region
FCγR: Fc region of human IgG
FTD: Frontotemporal dementia
FTLD: Frontotemporal lobar dementia
GABA: Gamma-aminobutyric acid
GFAP: Glial fibrillary acid protein
GSK3β: Glycogen synthase kinase 3 beta
GWAS: Genome wide association studies
H3Kme: Histone H3 lysine methylation (2, 3)
Hsp60: Heat shock protein 60
HSVTK: Herpes simplex virus-1 thymidine kinase
ICD: Intracellular domain
IFNγ: Interferon gamma
Ig: Immunoglobulin (G)
IL1β: Interleukin 1 beta
IL: Interleukin (4, 6, 13)
iNOS: Inducible nitric oxide synthase
ITAM: Immunoreceptor tyrosine-based activation motif
ITIM: Immunoreceptor tyrosine-based inhibitory motif
LAB: Linker for activation of B cells
LDL: Low-density lipoprotein
LOAD: Late onset Alzheimer’s disease
LPS: Lipopolysaccharides
MAF: Minor allele frequency
MAPK: Mitogen-activated protein kinase
MCAO: Middle cerebral artery occlusion
MCI: Mild cognitive impairment
MCP1: Monocyte chemoattractant protein-1
MCSF: Macrophage colony stimulating factor
MDL-1: Myeloid DAP12-associating lectin-1
MMP: Matrix metallopeptidase (2, 9)
MMSE: Mini-Mental State Examination
MS: Multiple sclerosis
MS4A: Membrane-spanning 4-domain family, subfamily A
N2A: Neuro 2A
NDDs: Neurodegenerative diseases
NFAT: Nuclear factor of activated T-cells
NfκB: Nuclear factor kappa-light-chain enhancer of activated B cells
NHD: Nasu-Hakola disease
Nkp44: Natural cytotoxicity triggering receptor 2
NO: Nitric oxide
P2RY12: Purinergic receptor P2Y, G-protein coupled, 12
PBMC: Peripheral blood mononuclear cell
PD: Parkinson’s disease
PGRN: Progranulin
PHF: Paired helical filament
PI3K: Phosphoinositide 3-kinase
PKC: Protein kinase C
PLCγ: Phospholipase C gamma
PLOSL: Polycystic lipomembraneous osteodysplasia with sclerosing leukoencephalopathy
PSEN: Presenilin
p-tau: Phosphorylated tau
Rac: Related to A and C protein kinases
RANKL: Receptor activator of nuclear factor kappa-B ligand
ROS: Reactive oxygen species
RXR: Retinoid X receptor
SAMP8: Senescence accelerated mouse-prone 8
SHP: SH-2 domain-containing protein phosphatase
SHIP1: SH-2 domain-containing inositol 5′ polyphosphatase 1
SIRPβ: Signal-regulatory protein beta
SNAP25: Synaptosomal-associated protein 25
SNPs: Single nucleotide polymorphisms
sTREM: Soluble triggering receptor expressed on myeloid cells (1, 2)
Syk: Spleen tyrosine kinase
TLR: Toll-like receptor
TM: Transmembrane
TNFα: Tumor necrosis factor alpha
TREM: Triggering receptor expressed on myeloid cells (1, 2, 3)
TREML: Triggering receptor expressed on myeloid cells-like transcript (1, 2, 3, 4, 6)
TUNEL: Terminal deoxynucleotidyl transferase dUTP nick end labeling
UTR: Untranslated region
Vav: Vav guanine nucleotide exchange factor
Wnt: Wingless-type MMTV integration site family
WT: Wild-type
YKL-40: Chitinase-3-like protein 1
